# Haplotype divergence and multiple candidate genes at *Rphq2*, a partial resistance QTL of barley to *Puccinia hordei*

**DOI:** 10.1007/s00122-015-2627-5

**Published:** 2015-11-05

**Authors:** F. K. S. Yeo, Y. Wang, T. Vozabova, C. Huneau, P. Leroy, B. Chalhoub, X. Q. Qi, R. E. Niks, T. C. Marcel

**Affiliations:** Laboratory of Plant Breeding, Wageningen University, Droevendaalsesteeg 1, 6708PB, 6700 AJ Wageningen, The Netherlands; Department of Plant Science and Environmental Ecology, Faculty of Resource Science and Technology, University Malaysia Sarawak, 94300 Kota Samarahan, Sarawak Malaysia; Key Laboratory of Plant Molecular Physiology, Institute of Botany, Chinese Academy of Sciences, Nanxincun 20, Fragrant Hill, Beijing, 100093 China; The Institute of Botany of the Academy of Science of the Czech Republic, Zámek 1, 252 43 Průhonice, Czech Republic; INRA, UMR1165, Unité de Recherche en Génomique Végétale, 91057 Evry, France; Université d’Evry Val d’Essonne, UMR1165, Unité de Recherche en Génomique Végétale, 91057 Evry, France; INRA, UMR1095, Genetics Diversity and Ecophysiology of Cereals, 63039 Clermont-Ferrand, France; Université Blaise Pascal, UMR1095, Genetics Diversity and Ecophysiology of Cereals, 63039 Clermont-Ferrand, France; INRA, UMR1290, BIOGER, 78850 Thiverval-Grignon, France; AgroParisTech, UMR1290, BIOGER, 78850 Thiverval-Grignon, France

## Abstract

*****Key message***:**

***Rphq2*, a minor gene for partial resistance to*Puccinia hordei*, was physically mapped in a 188** **kbp introgression with suppressed recombination between haplotypes of*rphq2*and*Rphq2*barley cultivars.**

**Abstract:**

Partial and non-host resistances to rust fungi in barley (*Hordeum vulgare*) may be based on pathogen-associated molecular pattern (PAMP)-triggered immunity. Understanding partial resistance may help to understand non-host resistance, and vice versa. We constructed two non-gridded BAC libraries from cultivar Vada and line SusPtrit. Vada is immune to non-adapted *Puccinia* rust fungi, and partially resistant to *P. hordei.* SusPtrit is susceptible to several non-adapted rust fungi, and has been used for mapping QTLs for non-host and partial resistance. The BAC libraries help to identify genes determining the natural variation for partial and non-host resistances of barley to rust fungi. A major-effect QTL, *Rphq2*, for partial resistance to *P. hordei* was mapped in a complete Vada and an incomplete SusPtrit contig. The physical distance between the markers flanking *Rphq2* was 195 Kbp in Vada and at least 226 Kbp in SusPtrit. This marker interval was predicted to contain 12 genes in either accession, of which only five genes were in common. The haplotypes represented by Vada and SusPtrit were found in 57 and 43 %, respectively, of a 194 barley accessions panel. The lack of homology between the two haplotypes probably explains the suppression of recombination in the *Rphq2* area and limit further genetic resolution in fine mapping. The possible candidate genes for *Rphq2* encode peroxidases, kinases and a member of seven-in-absentia protein family. This result suggests that *Rphq2* does not belong to the NB-LRR gene family and does not resemble any of the partial resistance genes cloned previously.

**Electronic supplementary material:**

The online version of this article (doi:10.1007/s00122-015-2627-5) contains supplementary material, which is available to authorized users.

## Introduction

Partial resistance is an incomplete host resistance which retards epidemic development despite a compatible infection type (Niks et al. 2011; Parlevliet and van Ommeren 1975). Partial resistance of barley to *Puccinia hordei* consists of the components lower infection frequency, lower sporulation rate and longer latency period of the pathogen, and such effects are not associated with hypersensitivity. Evidence suggests partial resistance of barley to powdery mildew and rust fungi to be a weak form of non-host resistance based on pathogen-associated molecular pattern (PAMP)-triggered immunity (PTI) (Jones and Dangl 2006; Niks and Marcel 2009; Niks et al. 2011; Trujillo et al. 2004). Non-host resistance is, by definition, a resistance observed in all genotypes of a plant species to all genotypes of a potential pathogen species (Niks et al. 2011). However, plant species may turn out to have a marginal host or near-non-host status to some pathogen species (Niks 1987). Barley (*Hordeum vulgare*) is such a marginal host or near-non-host to *Puccinia triticina*, *Puccinia hordei*-*murini* and some other non-adapted rust fungi because a small proportion of barley accessions (less than 10 %) are susceptible (>10 pustules on the first leaf) at seedling stage when inoculum is applied at high density (Atienza et al. 2004). In the context of this paper, non-adapted rust fungi are rust fungi that are poorly or not adapted to barley, but primarily to one or few other plant species. We try to investigate non-host resistance by dissecting the genetics of near-non-host resistance with the assumption that we can extrapolate the findings to explain also the basis of full non-host resistance. SusPtrit was developed for such a purpose by accumulating susceptibility genes from rare barley accessions that were somewhat susceptible to *P. triticina* at the seedling stage. SusPtrit is, at the seedling stage, highly susceptible to *P. triticina* and to at least nine other non-adapted rust fungi (Atienza et al. 2004). This experimental line was used to develop mapping populations Vada/SusPtrit (Jafary et al. 2006), Cebada Capa/SusPtrit (Jafary et al. 2008) and SusPtrit/Golden Promise (Yeo et al. 2014), as well as a set of near-isogenic lines for different resistances QTLs (Yeo et al. unpublished data). Resistance of barley to non-adapted rust fungi inherits polygenically just as partial resistance to *P. hordei*, the barley leaf rust fungus (Jafary et al. 2006, 2008; Marcel et al. 2007b; Niks et al. 2000; Qi et al. 1998).

The locations of genes for resistance to non-adapted rust fungi tend to overlap with those for partial resistance to *P. hordei* (González et al. 2010; Jafary et al. 2008; Niks and Marcel 2009) and they share the same prehaustorial resistance mechanism, viz. a high rate of failed attempt to haustorium formation (Niks 1983a, b). If both the partial resistance to adapted fungi and non-host resistance to non-adapted fungi are mainly based on PTI, a better understanding of partial resistance will help us to gain more insight into non-host resistance, and vice versa. More than 20 partial resistance quantitative trait loci (QTLs) to *P. hordei* have been mapped at seedling and adult plant stages (Jafary et al. 2006; Marcel et al. 2007b, 2008; Niks et al. 2000; Qi et al. 1998, 1999). To determine the underlying genes of those QTLs, we need to identify and validate the candidate genes of a selection of QTLs. To date, five large-effect resistance QTLs have been cloned (Fu et al. 2009; Fukuoka et al. 2009; Hayashi et al. 2010; Krattinger et al. 2009; Manosalva et al. 2009). The cloned QTLs are different from each other in gene structure and function, which implies a diversity of mechanisms underlying partial resistance. The genes that have been cloned so far do not have the typical modular NB-LRR structure of *R*-genes. *Rphq2* is one of the partial resistance QTLs to *P. hordei* mapped at the telomeric region of chromosome 2HL in L94/Vada (Qi et al. 1998), Vada/SusPtrit (Jafary et al. 2006) and in an association mapping study comprising 146 barley genotypes (Kraakman et al. 2006). In L94/Vada and Vada/SusPtrit populations, Vada is the donor of *Rphq2*. Vada is a cultivar developed from *Hordeum laevigatum*/Gold (Dros 1957). Near-isogenic lines (NILs) are available for *Rphq2* in L94 genetic background (L94-*Rphq2*) (Marcel et al. 2007a; van Berloo et al. 2001) and for *rphq2* of L94 in Vada background (Vada-*rphq2*) (Marcel et al. 2007a). L94 is an Ethiopian landrace-derived line that has some level of susceptibility to some non-adapted rust fungi (e.g., *Puccinia triticina, P. hordei*-*secalini* and *P. hordei*-*murini*) (Atienza et al. 2004; Niks 1983a). When L94-*Rphq2* was inoculated with *P. hordei*-*secalini* and *P. hordei*-*murini*, it had a significantly lower infection level compared to L94 (Yeo et al. unpublished data). This suggests that the postulated *Rphq2* gene explaining the resistance to *P. hordei* also affects the resistance to some non-adapted rust species. Recently, Johnston et al. (2013) suggested that *Rphq2* is possibly a weaker allelic form of a novel leaf rust resistance gene *Rph22* (or *Rph22.ak*) found in *H. bulbosum*, a non-host species for *P. hordei*. Similar to *Rphq2*, *Rph22* confers a non-hypersensitive reaction resistance. Therefore, it is interesting to clone *Rphq2* which will provide molecular information to further study partial and non-host resistances, and their possible association. The high-resolution mapping of *Rphq2* allowed pinpointing this QTL to an interval of about 0.1 cM, corresponding to about 121–198 kb (Marcel et al. 2007a). This estimated physical interval is sufficiently small to justify the development of a Bacterial Artificial Chromosomes (BAC) library to pin down *Rphq2* to one or few candidate genes.

Construction and organization of BAC libraries remains laborious and costly, especially for organisms with large and complex genomes like barley [4.98 Gb (The International Barley Genome Sequencing Consortium 2012)]. In barley, about 200,000 clones with an average insert size of 120 kb would be required to achieve a genome coverage of five genome-equivalents, which is needed for a more than 99 % probability of recovering any specific sequence of interest. To date, gridded BAC libraries are available for barley cv. Morex (Schulte et al. 2011; Yu et al. 2000), Haruna Nijo (Saisho et al. 2007) and a doubled haploid barley line CS134 derived from Clipper/Sahara-3771 (Shi et al. 2010). The inconveniences linked to the gridding, storage and maintenance of such a quantity of clones can be circumvented by the pooled library approach described by Ma et al. (2000) for wheat and Isidore et al. (2005) for barley. This approach consists of pooling several hundreds of clones together without the need of picking and storing individual clones. The first pooled BAC library of barley developed from cv. Cebada Capa was successfully used to establish a single contig of six BAC clones spanning 230 kb at the *Rph7* locus on chromosome 3H (Isidore et al. 2005). The BAC libraries from the four mentioned barley genotypes could help in the construction of physical maps around any target gene, but to isolate genes of interest in plants, it is often essential to construct BAC libraries from specific genotypes. Indeed, the gene content may vary between individuals of the same species and the gene of interest may not be present in the genomic libraries of conspecific accessions. This consideration is especially relevant concerning the genes involved in resistance to pathogens, which are known to be under strong selective pressure (Meyers et al. 2005; Salvaudon et al. 2008; Shen et al. 2006).

The current study aimed to develop two non-gridded BAC libraries from cultivar Vada and line SusPtrit, which will allow the isolation of genes for partial and non-host resistances. Having a BAC library from the resistant parent as well as from a susceptible parent is required because genes involved in such resistances can either be a resistance or susceptibility factor. After screening the newly developed BAC libraries, we identified and sequenced BAC clones in the *Rphq2* region of both genotypes. The assembly and annotation of BAC sequences revealed a major divergence in haplotypes at the *Rphq2* locus and several genes that might be responsible for the phenotypic contrast between Vada and SusPtrit for partial resistance due to *Rphq2*.

## Materials and methods

### Construction of two non-gridded BAC libraries from Vada and SusPtrit

We constructed two non-gridded BAC libraries of barley from the cv. Vada and from the experimental line SusPtrit, respectively. Vada not only carries the resistance allele of our target QTL for map-based cloning, *Rphq2*, but also many other QTLs for partial and non-host resistances to adapted and non-adapted rust species (Jafary et al. 2006, 2008), for which SusPtrit contains the susceptibility allele.

The methodology followed to construct the two BAC libraries has been described in detail by Peterson et al. (2000), with several modifications proposed in subsequent reports (Allouis et al. 2003; Chalhoub et al. 2004; Isidore et al. 2005). A detailed description of the methodology to construct the BAC libraries of Vada and SusPtrit is available as supplementary material (Supplemental Material 1). Briefly, high molecular weight (HMW) DNA was isolated from 40 to 50 g of 2–4-week-old leaves. Plugs of HMW DNA were partially digested with the *Hind*III restriction enzyme. Partially digested DNA was subjected to a single size selection and fractions of DNA fragments were recovered from agarose plugs in the ranges 50–100 kb (H0 fraction), 100–150 kb (H1 fraction), 150–200 kb (H2 fraction), 200–250 kb (H3 fraction) and 250–300 (H4 fraction). The insert DNA from H0 to H4 fractions was ligated separately into the pIndigoBAC vector (CalTech) prepared for high-efficiency cloning with *Hind*III as described by Chalhoub et al. (2004) or into the commercial pIndigoBAC-5 vector (Epicentre Biotechnologies). BAC clones were obtained by electroporation of the de-salted ligation mixtures into ElectroMax DH10B electrocompetent cells (Invitrogen). Pools of BAC clones were prepared by collecting colonies on plates calibrated to contain an average of about 1500 colonies per plate, as suggested by Isidore et al. (2005). The pools were then aliquoted into four tubes, each corresponding to one copy of the library (copies A, B, C and D). Copy A is stored in a −80 ^°^C freezer at Unité de Recherche en Génomique Végétale (URGV, Evry, France), copy B at Institute of Botany, Chinese Academy of Sciences (IBCAS, Beijing, China). Copies C and D are stored at Wageningen UR, Plant Breeding (WUR, the Netherlands).

### Characterization of the BAC libraries

Twenty-four BAC clones were randomly selected from the fractions H1, H2 and H3 of each library (i.e., 72 BAC clones per library) and grown for 24 h at 37 °C in 1.5 ml LB medium containing 12.5 μg CAM. The BAC DNA was extracted following an alkaline lysis procedure (Sambrook et al. 1989) with ready-to-use buffers P1, P2 and P3 (Qiagen) and digested overnight with *Not*I (New England Biolabs). Digested products were separated on a 1 % SeaKem^®^ LE agarose gel (BMA) in 0.5× TBE in a CHEF-DR™ II apparatus (Bio-Rad) with the following pulsed field gel electrophoresis parameters: 200 V, 5–15 s switch time, for 14.3 h at 10 °C. The insert sizes of selected BAC clones were estimated after comparison with the CHEF DNA size standard lambda ladder (Bio-Rad) run in the same gel.

BAC pool DNA was isolated from 250 μl aliquot per pool from the copy D of the libraries as described previously. The two barley BAC libraries were characterized for genome representation by PCR screening of 46 pools per library with one microsatellite marker from each of the 14 barley chromosome arms. The markers were selected from the barley microsatellite consensus map of Varshney et al. (2007). Robust markers that were polymorphic between Vada and SusPtrit were selected in priority. For only two microsatellites, GBMS062 and GBM1482, Vada and SusPtrit had the same allele. The number of positive pools was determined by counting the number of pools displaying a band of similar size as the one of the parental genomic DNA run on the same gel. None of the bands amplified in a BAC pool from one genotype had the size of the allele from the other genotype, indicating that contamination of one library with clones from the other library is unlikely. The reverse primer of each microsatellite was labeled with IRDye-700 or IRDye-800 and the PCR product visualized on a LICOR 4200 DNA sequencer (LICOR^®^ Biosciences).

### Identification of BAC clones spanning the *Rphq2* locus

The work flow for identifying Vada and SusPtrit BAC clones spanning the *Rphq2* locus is presented in Supplemental Fig. 1. The BAC libraries were screened following a PCR-based method. In a first step, to screen the BAC pools and to identify positive BAC clones, we used molecular markers known to be closely linked to *Rphq2* (Marcel et al. 2007a; Table 1). In a second step, after the identification and sequencing of several positive BAC clones, we used primers designed to amplify the BAC end sequences (bes), the genes annotated in these clones and newly developed markers. We also used primers to amplify sequences at the edges of gaps in the already assembled BAC sequences (Table 1). Positive single-BAC clones were validated after PCR amplification with the markers and primers used during the screening process and maintained in glycerol stock (LB supplemented with 25 % glycerol). The insert size of the BAC clones was determined as described in the previous section before further analyses. The BAC clones were named as follows: “BAC pool number; Sub-pool number (384-well plate number); row letter; column number; single-colony letter” (e.g., V41P7L3A).

**Fig. 1:**
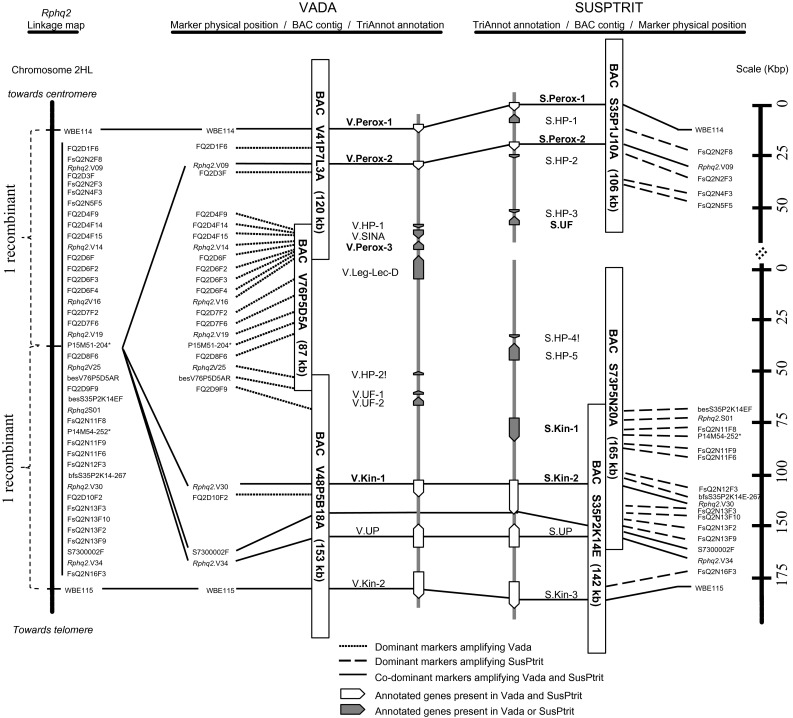
Representation of the 0.1 cM marker interval between WBE114 and WBE115 containing *Rphq2*: including the linkage map, the BAC contig of Vada and SusPtrit, and the genes annotated with the TriAnnot pipeline. The size of the BAC clones was estimated by *NotI* digestion. The genes followed by ! have been predicted only by the TriAnnot pipeline and not by RiceGAAS. The genes in bold were supported by sequences from the transcriptomes of Vada and L94, indicating that they are expressed in the leaves. Markers followed by * are AFLP-converted single locus PCR markers developed by Marcel et al. (2007a). *HP* hypothetical protein, *Kin* kinase, *Leg-Lec-D* legume lectin domain, *Perox* peroxidase, *S* SusPtrit, *SINA* seven-in-absentia protein, *UF* unknown function, *UP* uncharacterized protein, *V* Vada

**Table 1:** Primer pairs used to identify BAC clones of Vada and SusPtrit at the *Rphq2* locus

Name	Primers sequences (5′–3′)	*T*_a_ (°C)
^M^WBE114^*VS*^	*F*: GGCGACCTCCAGCGTATC	58
	*R*: GTGGTTCGGTCCTTGATGAG	
^M^WBE115^*VS*^	*F*: GGCGGTCGGCATCGTCCAGT	61
	*R*: ATGCGTCCACAAAACCAATCTTCA	
^M^P15M51-204^*V*^	*F*: CGGAGGAAACATGGACAACGAA	56
	*R*: AGCGAGCTCACTGCCAATCTACC	
^M^P14M54-252^*S*^	*F*: AGACCAGCATTACCTAAGCAGAGA	56
	*R*: AGAGGAGAGTGAGTGTAGGTGTCG	
^M^besV76P5D5AR^*V*^	*F*: GAGGAGCCGTGTCGTCTTGT	56
	*R*: CCGTTTCCGTTCACTGGTTAT	
^M^besS35P2K14EF^*S*^	*F*: TTGAAACAGCTGGGGTCTT	58
	*R*: TGGTACACAAATATTCGTCTGC	
^MG^ *Rphq2*.S01^*S*^	*F*: TGAAGGCGGGTTTGGTGTGGTGTA	58
	*R*: CCCGCGTATGATTCTCTGCCTCTT	
^MG^ *Rphq2*.V30^*S*^	*F*: CGGCGGTGCGATCATAGAAT	65
	*R*: TCCCCGGCCGTAGAGTCC	
^G^ *Rphq2*.V32^*S*^	*F*: GGGGCCCCGGCTATCGTGTA	65
	*R*: AACTTTCCGCGGCAATCCTTCTTCT	
^a^S35P100001F4^*S*^	*F:* CCTCGCTAGTCAAGGAGGTG	65
	*R:* GTGGCTGTTGTAGGGACGAT	
^a^S35P100004F2^*S*^	*F:* TTAATTTCTGCTCGCGTGTG	65
	*R:* TGCATGCACTCCTCGTTTAG	
^M^S7300002F^*S*^	*F:* GACGTTGAGGAGAGCAAAGG	65
	*R:* GCCGTTTATCACGAGGTTGT	

*T*_a_ is the annealing temperature

^M^A molecular marker

^G^An annotated gene

^MG^An annotated gene converted into a marker

^a^A short DNA fragment at the edge of a gap in assembled BAC sequences

^*S*^Primer pair used to screen only the BAC library of SusPtrit

^*V*^Primer pair used to screen only the BAC library of Vada

^*VS*^Primer pair used to screen the BAC library of Vada as well as SusPtrit

All the confirmed positive BAC clones from the Vada and SusPtrit libraries were fingerprinted and their extremities sequenced. BAC clone fingerprints were obtained following the AFLP procedure from Brugmans et al. (2006) using the H*ind*III/*Taq*I restriction enzyme combination. The generated fragments were separated on a LICOR 4200 DNA sequencer (LICOR Biosciences, Lincoln, NE, USA). Shared bands between BAC clones indicated sequence overlaps between the clones. BAC end sequences were obtained following a procedure consisting of a nested PCR approach on restriction digests of BAC clones DNA ligated with specific adaptors (Supplemental Material 1). Primers which amplify the BAC ends were designed using Lasergene software (DNASTAR^®^ 8 Inc., Madison, WI, USA).

### BAC clone sequencing and annotation

The *Rphq2* genetic window is delimited by proximal (WBE114) and distal (WBE115) markers for which at least one recombination event has been found with *Rphq2*. Three BAC clones which fully covered the *Rphq2* genetic window (V41P7L3A, V48P5B18A and V76P5D5A) were sequenced following a Sanger approach at Macrogen. One SusPtrit BAC clone was sequenced via a Sanger approach as well, S35P2K14E. The number of reads obtained for the BAC clones of Vada and SusPtrit ranges from 1000 to 1740 reads with an average length of 867 bp, corresponding to 8× sequencing depths. For each BAC clone, the short Sanger sequences were assembled in contigs and ordered by Macrogen. Several gaps remained within the BAC clones with 5–14 contigs per clone. Therefore, the three BAC clones of Vada and S35P2K14E of SusPtrit were re-sequenced following a 454 sequencing approach at Greenomics™ to bridge the gaps. Two additional BAC clones of SusPtrit (S35P1J10A and S73P5N20A) were also sequenced following this 454 sequencing approach. The three SusPtrit BAC clones, S35P2K14E with S35P1J10A and S73P5N20A, together cover only partially the targeted *Rphq2* genetic window. The number of reads per BAC clone obtained from 454 sequencing ranges from 15,570 to 38,120 reads with an average length of 350 bp, corresponding to 20× sequencing depths for each BAC clone. For each BAC clone, the short sequences obtained by 454 sequencing were assembled in contigs by Greenomics™.

The obtained 454 sequence contigs were aligned to the previously assembled Sanger sequence contigs [Lasergene software (DNASTAR^®^ 8 Inc., Madison, WI, USA)]. No discrepancy was observed between the assemblies obtained via both approaches. Based on the AFLP fingerprints of the BAC clones of Vada, V76P5D5A overlaps proximally with V41P7L3A and distally with V48P5B18A. We identified the overlapping sequences and confirmed the fingerprints. According to the AFLP fingerprints of the BAC clones of SusPtrit, S73P5N20A overlaps with S35P2K14E, and S35P1J10A is located proximally but is not overlapping. We identified the overlapping sequences between S73P5N20A and S35P2K14E which also helped us to order the 454 sequence contigs. The 454 sequence contigs obtained for clone S35P1J10A were ordered according to a dot plot analysis against V41P7L3A which shares the same marker (WBE114). The dot plot analysis was carried out using MUMmer software (Kurtz et al. 2004).

The consensus sequence corresponding to the *Rphq2* genetic window of Vada, flanked by markers WBE114 and WBE115, was generated and annotated using the TriAnnot pipeline version 3.5 (Leroy et al. 2012) following the barley default analysis template (http://clermont.inra.fr/triannot). The consensus sequence corresponding to the *Rphq2* genetic window of SusPtrit was annotated separately using the same pipeline, TriAnnot_v3.5. The sequences of Vada and SusPtrit were also annotated separately using RiceGAAS (Sakata et al. 2002; http://ricegaas.dna.affrc.go.jp/). Since the RiceGAAS is dedicated to rice and not barley, there is a chance for false positives when applied to barley sequences. Therefore, the sequence of the predicted genes were used to blast against the barley expressed sequence tag (EST) tentative consensus (TC) sequences from the barley TIGR Gene Indices database (http://www.tigr.org/tdb/tgi/index.shtml). A predicted gene was retained if the best blast hit was above a threshold e value ≤1.0E−15. Comparison was made between the gene annotations by TriAnnot_v3.5 and RiceGAAS pipelines.

### Transcriptome sequencing

The results from the annotation were compared with transcriptome data obtained from leaf tissue of L94 and Vada. Tissue was collected from the first leaf of seedlings and from flag leaves of L94 and Vada at 0, 6, 12 and 48 h after inoculation with *P. hordei*. The samples were processed by the Beijing Genomics Institute (http://www.genomics.cn/en/index). The RNA isolation and cDNA synthesis were performed according to the TruSeq™ RNA sample preparation guide from Illumina (Catalog # RS-930-20 01, Part # 15008136 Rev. A, November 2010). After extraction, RNA samples were combined in aliquots to obtain two pools of leaf RNA, one for L94 and one for Vada. The cDNA libraries were constructed for each pool and sequenced on an Illumina HiSeq™iSeqq. The software TopHat was used to map the raw reads of transcriptome data on the previously assembled BAC contig sequences from Vada and SusPtrit. The mapping results were used to improve the annotation of the predicted genes and to obtain information on their expression in infected and non-infected leaf tissues.

### Marker saturation of the regions containing *Rphq2*

Between the flanking markers (WBE114 and WBE115), BAC end sequences and annotated gene sequences were used to develop sequence characterized amplified regions (SCAR) and cleaved amplified polymorphic sequence (CAPS) markers, polymorphic between Vada, SusPtrit and L94. Markers polymorphic between Vada and L94 were genetically mapped using the homozygous recombinant plants from Marcel et al. (2007a) to confirm their position and order. SCAR and CAPS markers were also developed from the AFLP fingerprints by converting polymorphic bands to single locus PCR markers following the strategy proposed by Brugmans et al. (2003). Primers were designed using Lasergene software (DNASTAR^®^ 8 Inc., Madison, WI, USA). The markers developed were used to assist in ordering the BAC clones.

WBE114 and WBE115 (Table 1) together with two newly developed markers, viz. *Rphq2*.S01 (Table 1) and *Rphq2*.V09; F: 5′-GCCTCTACTTCCACGACTGC-3′, R: 5′-CCGGAGATGAC GATGATGT-3′) were used to screen the Morex BAC library (Nils Stein, Leibniz Institute of Plant Genetic and Crop Plant Research, IPK) to identify the homologous *Rphq2* region in the Morex genomic sequence.

### Haplotypes of one Vada-specific and two SusPtrit-specific genes in barley accessions

A large part of the sequence bracketed by WBE114 and WBE115 had little or no homology between Vada and SusPtrit, and contained several cultivar-specific genes. We determined the occurrence of one Vada-specific (V.Perox-3) and two SusPtrit-specific genes (S.UF and S.Kin-1) in 194 barley accessions, including Vada and SusPtrit. These three genes were expressed in leaf tissue and might be considered candidate genes for the *Rphq2* gene. Most (120) of the accessions had been used in an association study by Kraakman et al. (2006), but we added 74 accessions. Those additional accessions came from a panel of barley accessions previously used to study non-host resistance (Atienza et al. 2004), or were used to generate mapping populations (like Golden Promise and Steptoe) or featured as important ancestors of European barley cultivars. Primer pairs with specific amplification for the Vada- and SusPtrit-specific genes were designed using DNAMAN (Table 2). A primer pair was designed to amplify part of an exon and part of an intron to ensure specific amplifications.

**Table 2:** Primer pairs for specific amplification of selected Vada- and SusPtrit-specific genes

Name	Gene	Line	Primers sequences (5′–3′)	*T*_a_ (°C)^a^
V.Perox-3	Peroxidase	Vada	*F*: CGTATGGGTTTGTAGGTGTAGCAT	60.4
			*R*: CAGTTCGCCAAGTCGATGACCA	62.8
S.UF	Unknown function	SusPtrit	*F*: CCGAGATCCTTGTTGCACTATTAC	59.4
			*R*: GGTATACCTGTCACTAACAAACACT	58.7
S.Kin-1	Kinase	SusPtrit	*F*: CCGGTACAGTCCATGTTTTTCTC	59.5
			*R*: CTCAGTGCTTCAGATGTTGCTTAG	60.0

^a^ *T*_a_ (°C) annealing temperature

A haplotype of a barley accession is based on the presence (P) and absence (A) of V.Perox-3, S.UF and S.Kin-1. A haplotype of ‘PAA’ indicates the presence of V.Perox-3 and the absence of S.UF and S.Kin-1; ‘APP’ the absence of V.Perox-3 and the presence of S.UF and S.Kin-1.

### Comparative mapping in barley, rice and *Brachypodium*

The sequence of annotated genes in the refined region containing *Rphq2* was used for blast searches of rice and *Brachypodium distachyon* homologous genes, in the Rice Genome Annotation Project blast search (http://rice.plantbiology.msu.edu/analyses_search_blast.shtml) and in the *B. distachyon* blast portal (http://blast.brachypodium.org/), respectively. For each annotated gene in barley, the best blast hit was retained above a threshold *e* value ≤1.0E−15.

## Results

### Construction and characterization of the BAC libraries

The Vada library was organized in 116 pools named V1 to V116 (Supplemental Table 1), containing an average of 1435 clones per pool. Based on the count of blue (non-recombinant) and white (recombinant) colonies per plate, the percentage of recombinant clones was estimated to be 96.8 %. Thus, the library consists of approximately 161,000 recombinant clones. The average size of inserts ranges from 67 to 98 Kbp. The observed insert sizes for each fraction do not correspond well to the expectations based on the size selection (Supplemental Table 2).

The SusPtrit library was organized in 110 pools named S1 to S110 (Supplemental Table 3), containing an average of 1606 clones per pool. The percentage of recombinant clones was estimated to be 97.9 % based on the count of blue and white colonies per plate. Thus, the library consists of approximately 173,000 recombinants clones. The average size of SusPtrit inserts ranged from 107 Kbp for selected fraction H1–141 Kbp for selected fraction H3 (Supplemental Table 4).

Based on a haploid barley genome size of 4.98 Gb (The International Barley Genome Sequencing Consortium 2012) and on the genome coverage of each fraction of the libraries (Supplemental Tables 2 and 4), we estimated that the coverage of the Vada and SusPtrit BAC libraries are approximately 2.6 and 3.7 genome-equivalents, respectively. Together, the libraries cover 6.4 genome-equivalents that allow for a probability greater than 99 % of recovering any specific sequence from the barley genome (Clarke and Carbon 1976).

To verify the genome representation of the libraries, we screened 46 pools of Vada (V1–V46) and 46 pools of SusPtrit (S1 to S46) corresponding approximately to 1.4 and 1.9 genome-equivalents, respectively, with 14 microsatellite markers (Supplemental Table 5). An average of 2.7 positive pools per microsatellite marker was obtained for the Vada library and an average of 3.5 positive pools for the SusPtrit library (Supplemental Table 5). All markers were represented at least once in the 46 pools of the SusPtrit library and only two markers were not represented in the 46 pools of the Vada library, indicating that the overall barley genome is well represented in our BAC libraries. Based on the average representation of the 14 microsatellite markers in 46 pools per library, we estimated that the total coverage of the Vada and SusPtrit BAC libraries are 5.0 and 6.8 genome-equivalents, respectively.

### Generation of Vada and SusPtrit sequences at *Rphq2*

We used four primer pairs for the BAC library of Vada and ten primer pairs for the BAC library of SusPtrit (Table 1) to screen for BAC clones spanning the Vada or SusPtrit allele at *Rphq2*. For Vada, the four primer pairs detected 16 positive BAC pools in total from the Vada BAC library and for SusPtrit the ten primer pairs detected a total of 21 positive BAC pools in the SusPtrit BAC library (Supplemental Table 6). The BAC pools positive for two or more primer pairs were prioritized for the identification of single positive BAC clones. Finally, we validated seven Vada BAC clones originating from four Vada BAC pools and 17 SusPtrit BAC clones originating from nine SusPtrit BAC pools. All BAC clones were fingerprinted. The order of the BAC clones was not only based on the BAC fingerprint, but also on the primer amplification (Supplemental Table 7).

From the seven BAC clones of Vada, the AFLP fingerprint revealed a minimum tiling path of only three clones [V41P7L3A (120 Kbp), V76P5D5A (87 Kbp) and V48P5B18A (150 Kbp)], which were overlapping each other to cover the *Rphq2* genetic window between markers WBE114 and WBE115 (Table 3). Those three BAC clones were sequenced following both Sanger and 454 sequencing approaches. The sequences obtained with one or the other approaches were assembled independently. The sequence assembly obtained from Sanger sequencing had ten gaps between WBE114 and WBE115, while the sequence assembly obtained from 454 sequencing had 12 gaps. Aligning both assemblies led to a consensus sequence with only two gaps left, one in the sequence of V76P5D5A and the other one in the sequence of V48P5B18A. Together, the two gaps represent approximately 7 Kbp, calculated by comparing the estimated insert size of each BAC clone with the size of the consensus sequence. The physical length of the three contigs covering the Vada BAC sequence from WBE114 to WBE115 is approximately 195 kb excluding the gaps.

**Table 3:** Ordering of the BAC clones from Vada (A) and SusPtrit (B) libraries according to positive PCR amplification with developed primers at the *Rphq2* locus

(A)	besV41P7L3AR	WBE114	besV76P5D5AF	besV41P7L3AF	P51M51-204	besV48P5B18AR	besV76P5D5AR	WBE115	besV48P5B18AF
V41P7L3A	+	+	+	+	−	−	−	−	−
V76P5D5A	−	−	+	+	+	+	+	−	−
V48P5B18A	−	−	−	−	−	+	+	+	+

(B)	besS35P1J10AR	WBE114	besS35P1J10AF	besS35P2K14EF	*Rphq2*.S01	P14M54-252	besS81P2C6AF	bfsS35P2K14EF-267	bfsS35P2K14EF-283	WBE115	besS7P2C21EF	bfsS35P2K14EF-468	besS35P2K14ER	besS81P2C6AR
S35P1J10A	+	+	+	−	−	−	−	−	−	−	−	−	−	−
S73P5N20A^a^	−	−	−	+	+	NT	NT	+	+	NT	NT	−	−	NT
S35P2K14E	−	−	−	+	−	+	+	NT	+	+	+	+	+	–
S7P2C21A	−	−	−	−	NT	−	−	NT	–	−	−	−	−	−
S81P2C6A	−	−	−	−	NT	−	+	NT	+	−	−	−	+	+

From the 17 BAC clones of SusPtrit, six had an identical AFLP fingerprint with S35P1J10A (105 Kbp) which harbored WBE114, but were not overlapping with any of the other identified SusPtrit BAC clones. On the other side, three BAC clones positive for WBE115 were overlapping with each other [S35P2K14E (140 Kbp), S7P2C21A (80 Kpb) and S81P2C6A (135 Kbp)], and with a fourth BAC clone [S73P5N20A (165 Kbp)] (Table 3). S35P2K14E was selected for sequencing because it was positive not only for WBE115 but also for P14M54-252, a marker mapped closer to *Rphq2* (Marcel et al. 2007a; Fig. 1). S73P5N20A was also selected for sequencing because it overlapped with S35P2K14E distal to WBE115, and was expected to extend further into the *Rphq2* genetic window. The BAC clone S35P2K14E was sequenced following both Sanger and 454 sequencing approaches while the other two BAC clones S73P5N20A and S35P1J10A were sequenced following a 454 sequencing approach only. The sequences obtained with one or the other approaches were assembled independently. The sequence assembly of S35P2K14E from Sanger sequencing had six gaps, while the sequence assembly obtained from 454 sequencing for this clone had four gaps. Aligning both assemblies led to a consensus sequence of 122 Kbp with no gap for S35P2K14E. The sequence assemblies for the other two SusPtrit clones were composed of four contigs each (three gaps for each clone). The consensus sequence of the overlapping BAC clones S73P5N20A and S35P2K14E resulted in three contigs of sizes 139 Kbp, 563 bp, and 60 Kbp. The length of the available consensus SusPtrit sequence between WBE114 and WBE115 is 226 Kbp. But the SusPtrit DNA stretch going from WBE114 to WBE115 shall be slightly longer as the SusPtrit contig was not entirely covered with overlapping BAC clones.

### Sequence annotation

The pipeline TriAnnot_v3.5 (Leroy et al. 2012) predicted 12 genes on both Vada and SusPtrit contigs. The RiceGAAS pipeline predicted 33 genes for the Vada contig and 50 for the SusPtrit contig. Among the 33 predicted genes for Vada, 22 hit a barley EST in TIGR Gene Indices database above a threshold *e* value ≤1.0E−15. Among the 50 predicted genes for SusPtrit, 34 had a hit above the same threshold. In Vada as well as in SusPtrit 11 genes were common to the predictions of both TriAnnot_v3.5 and RiceGAAS pipelines.

The pipeline TriAnnot_v3.5 provided a more reliable prediction due to its specificity and sensitivity compared to RiceGAAS (Leroy et al. 2012). This is because RiceGAAS depends solely on ab initio predictions and was trained for annotating rice sequences rather than barley sequences. The pipeline TriAnnot_v3.5 predicts genes by combining ab initio prediction and biological evidence based on sequence similarity with known coding sequences (transcripts/proteins) from grass genomes, and the specific barley default setting was used for the analysis (version v3.5). Consequently, we present in detail the genes predicted for the Vada and SusPtrit contigs using TriAnnot_v3.5. For the Vada contig, the predicted genes encoded three peroxidases (V.Perox-1, -2, -3), two kinases (V.Kin-1, -2), one seven-in-absentia protein family member (V.SINA), one protein with a legume lectin domain (V.Leg-Lec-D), and five proteins with unknown function/or hypothetical/or uncharacterized (V.UF/HP/UP). Among the twelve predicted genes from the Vada sequence, V.Perox-1, -2, -3 and V.Kin-1 were supported by sequences from the transcriptome data of Vada, indicating their expression in leaf tissue. For the SusPtrit contig, the predicted genes encoded two peroxidases (S.Perox-1, -2), three kinases (S.Kin-1, -2, -3), and seven proteins with unknown function or hypothetical or uncharacterized (S.UF/HP/UP). Among the twelve predicted genes from SusPtrit sequence, S.Perox-1, -2, S.UF and S.Kin-1 and -2 were supported by sequences from transcriptome data of L94. The comparison of genes predicted in both cultivars indicated that V.Perox-1, V.Perox-2, V.UP, V.Kin-1 and V.Kin-2 from Vada were positioned at the same locus (allelic) as S.Perox-1, S.Perox-2, S.UP, S.Kin-2 and S.Kin-3 from SusPtrit, respectively. Their positions were supported by co-dominant markers (Fig. 1). The common genes between Vada and SusPtrit shared at least 99 % sequence similarity except for V.Kin-1 and S.Kin-2 which had only 90 % sequence similarity. The other annotated genes were not shared between Vada and SusPtrit (Fig. 1). The V.UF and V.HP from Vada did not align to the S.UF and S.HP from SusPtrit, respectively, and were regarded as different loci. The RiceGAAS pipeline predicted one additional kinase in SusPtrit. That sequence hit a barley EST in TIGR Gene Indices database with an *e* value of 1.0E−34. The likelihood of genes listed in Fig. 1 to be candidates for *Rphq2* will be discussed in the “Discussion”.

**Fig. 2:**
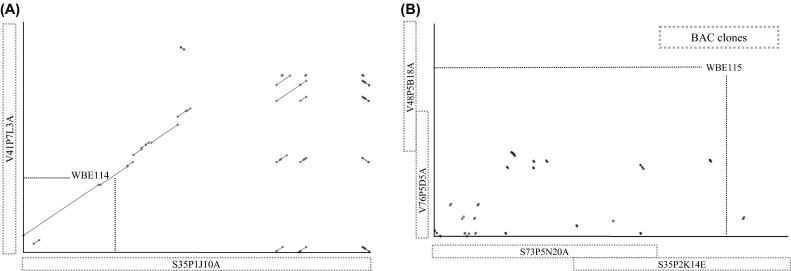
*Dot plots* suggesting lack of homology between the sequences of Vada and SusPtrit at *Rphq2*.* Red dots* indicate the sequence similarity between Vada and SusPtrit with the same orientation. *Blue dots* indicate the sequence similarity but either the Vada or SusPtrit sequence is inverted. **a** Alignment between the sequences of Vada and SusPtrit BAC clones proximal to the gap in SusPtrit BAC contig. **b** Alignment between the sequences of Vada and SusPtrit BAC clones distal to the gap in SusPtrit BAC contig (color figure online)

Using the sequence information between WBE114 and WBE115, we developed 39 new markers that were polymorphic between Vada and SusPtrit, and also determined their alleles for the line L94 carrying the susceptibility allele at *Rphq2*. Interestingly, SusPtrit and L94 have for all those markers the same allele. Together with two AFLP-converted single locus PCR markers developed by Marcel et al. (2007a), 41 markers were mapped between the flanking markers WBE114 and WBE115 (Supplemental Table 8). Among the 41 markers, 20 were dominant markers amplifying only Vada DNA and 17 were dominant markers amplifying only SusPtrit DNA (Fig. 1). Since the markers were developed by amplifying whole genome DNA, the dominance of the markers indicates that the sequences were Vada- and SusPtrit specific, respectively. There were four co-dominant markers; three were developed based on the common annotated genes between Vada and SusPtrit and one based on a random sequence from one of the sequence contigs of S73P5N20A. According to the sequence annotation and to the dominant nature of most markers located between WBE114 and WBE115, there seems to be a lack of homology between Vada and SusPtrit for the region containing *Rphq2*. This is further supported by a dot plot analysis (MUMmer) which compares the sequence of Vada and SusPtrit between WBE114 and WBE115 (Fig. 2). Sequence similarity is only observed on approximately the first 36 Kbp of S35P1J10A which includes WBE114.

### Homology of the region with other barley germplasm

We searched in the Morex genomic sequence (The International Barley Genome Sequencing Consortium 2012) the sequence corresponding to the divergent *Rphq2* region between Vada and SusPtrit. Two pairs of primers, respectively, amplifying the predicted genes V.Perox-2 (*Rphq2*.V09) and S.Kin-1 (Rphq2.S01), detected five overlapping BAC clones from Morex covering the *Rphq2* region (HVVMRXALLEA0011E05, HVVMRXALLEA0179K22, HVVMRXALLeA 0269J17, HVVMRXALLeA0278A17, and HVVMRXALLeA0299N24). The BAC clones are positioned between 806 and 1101 kb of contig 44195 (1.9 Mb) on chromosome 2HL (http://mips.helmholtz-muenchen.de/plant/barley/index.jsp). A total of 118 Morex sequence contigs (Mor_cont) from the BAC contig 44195 (kindly provided by Nils Stein, Leibniz Institute of Plant Genetic and Crop Plant Research, IPK) were aligned to the sequence between WBE114 and WBE115 of Vada and SusPtrit using Lasergene software with a minimum sequence match of 80 % (DNASTAR^®^ 8 Inc., Madison, WI, USA). Eleven of the 118 Mor_cont from BAC contig 44195 aligned to the Vada sequences and 17 to the SusPtrit sequences (Table 4). We found that Mor_cont 43090 was approximately 300 bp distal from WBE114, and that Mor_cont 2546833 aligned to a region including WBE115. We estimated the physical distance between WBE114 and WBE115 of Morex to be of approximately 254 kb, which is 28 kb longer than the estimated physical distance in SusPtrit. More sequences from SusPtrit than from Vada could be aligned to the Morex sequences, indicating that SusPtrit and Morex are likely to be more similar than Vada and Morex at the *Rphq2* locus.

**Table 4:** Alignment of 18 Morex sequence contigs on Vada and SusPtrit sequence assemblies between WBE114 and WBE115 (corresponding to Morex BAC contig 44195)

Morex contigs; (sequence size, bp)	Align to Vada	Align to SusPtrit
Mor_cont 43090; (5993)/WBE114^a^	Yes^b^	Yes
Mor_cont 53633; (3569)	Yes	No
Mor_cont 2267159; (240)	No	Yes
Mor_cont 88428; (1710)	No	Yes
Mor_cont 2550490; (9192)	No	Yes
Mor_cont 2343918; (327)	No	Yes
Mor_cont 41082; (7623)	No	Yes
Mor_cont 442275; (1129)	No	Yes
Mor_cont 280043; (1061)	No	Yes
Mor_cont 1590687; (2251)	Yes	Yes
Mor_cont 287733; (1989)	Yes	Yes
Mor_cont 224400; (1060)	Yes	Yes
Mor_cont 321571; (308)	Yes	Yes
Mor_cont 60124; (3413)	Yes	Yes
Mor_cont 1588307; (1769)	Yes	Yes
Mor_cont 1572547; (2594)	Yes	Yes
Mor_cont 8886; (1904)	Yes	Yes
Mor_cont 2546833; (3163)/WBE115^c^	Yes	Yes

^a^WBE114 is 301 bp distal from Mor_cont 43090

^b^Yes means that the Morex sequence aligned to the subject with a minimum sequence match of 80 %

^c^The WBE115 sequence of Vada aligned to Mor_cont 2546833 with one single nucleotide polymorphism and two for the WBE115 sequence of SusPtrit

We also developed specific primers to amplify V.Perox-3 (Vada specific), S.UF and S.Kin-1 (SusPtrit specific) to determine whether these genes were present in 194 barley accessions. Based on the presence (P) and absence (A) of the three genes, all the barley accessions can be grouped into two about equally common haplotypes viz., PAA (111 accessions, 57 %, including Vada and *H. laevigatum*) and APP (83 accessions, 43 %, including SusPtrit, Morex and L94) (Supplemental Table 9). We should note that “presence” of a gene does not imply that the accessions have identical sequences for the gene, and that “absence” refers to the lack of amplification. An accession for which a gene is scored as “absent” may have some version of the gene, but with too much sequence difference for the primers to amplify the DNA.

### The synteny between barley, rice and *Brachypodium*

The *Rphq2* region is located in the barley 2L1.0 region, which has a syntenic relationship with a region on rice Chromosome 4 (Marcel et al. 2007a) and a region on *Brachypodium* Chromosome 5 (Mayer et al. 2011). Homologs of the two genes used to develop markers WBE114 and WBE115 have indeed been identified on rice Chromosome 4 and on *Brachypodium* Chromosome 5 (Table 5). The interval between the rice homologs Loc_Os04g59260 and Loc_Os04g59320 contains five annotated genes encoding a hypothetical protein, two retrotransposon proteins, a strictosidine synthase, and a phospholipase C. The interval between the *Brachypodium* homologs Bradi5g27210 and Bradi5g27240 contains 2 genes encoding a peroxidase and a phospholipase C. The gene used to develop the marker WBE114 encodes a peroxidase precursor. Interestingly, this gene is present in a single copy in rice, in two copies in *Brachypodium* and in barley line SusPtrit, and in three copies in barley cultivar Vada (Table 5). In addition, only two additional genes seem to be shared between the three species and the two barley genotypes, i.e., a phospholipase C and a protein kinase used to develop marker WBE115. The sequence of the phospholipase C genes in rice and *Brachypodium* are highly homologous to the genes encoding the uncharacterized proteins V.UP and S.UP (Table 5). Therefore, V.UP and S.UP may correspond to phospholipase C genes. The remaining seven genes predicted only from Vada and seven genes predicted only from SusPtrit have no homolog in the identified synthetic regions of rice and *Brachypodium*. Nevertheless, some of those genes had a blast hit with value ≤1.0E−15 with rice or *Brachypodium* genes located outside of the *Rphq2* syntenic interval (Table 5).

**Table 5:** Best blast hits (≤1.0E−15) of the predicted genes in Vada and SusPtrit sequences with the rice and the *Brachypodium* gene catalogs

Vada	Rice	*Brachypodium*	SusPtrit	Rice	*Brachypodium*
V.Perox-1^a^	Loc_Os04g59260 (5.6E−127)	Bradi5g27210 (1.3E−134)	S.Perox-1^a^	Loc_Os04g59260 (3.4E−128)	Bradi5g27210 (2.7E−134)
Na			S.HP-1	x	x
V.Perox-2	Loc_Os04g59260 (3.9E−103)	Bradi5g27220 (9.7E−119)	S.Perox-2	Loc_Os04g59260 (1.0E−103)	Bradi5g27220 (3.0E−119)
Na			S.HP-2	x	x
Na			S.HP-3	x	x
Na			S.UF	LOC_Os07g35310 (4.2E−61)	Bradi1g25552 (3.0E−73)
V.HP-1	x	x	Na		
V.SINA	LOC_Os05g06070 (1.7E−32)	Bradi2g01770 (7.7E−43)	Na		
V.Perox-3	Loc_Os04g59260 (3.8E−108)	Bradi5g27220 (4.1E−120)	Na		
V.Leg-Lec-D	LOC_Os08g03002 (4.4E−60)	Bradi1g28320 (7.2E−63)	Na		
Na			S.HP-4	LOC_Os11g30140 (1.7E−24)	x
Na			S.HP-5	x	x
V.HP-2	x	x	Na		
V.UF-1	LOC_Os10g37260 (1.4E−66)	Bradi3g30820 (4.4E−63)	Na		
V.UF-2	LOC_Os10g37260 (4.0E−28)	Bradi3g30820 (1.2E−20)	Na		
Na			S.Kin-1	LOC_Os07g35310 (1.4E−136)	Bradi1g25552 (5.0E−194)
V.Kin-1	LOC_Os08g03020 (5.4E−140)	Bradi1g28320 (8.7E−225)	S.Kin-2	LOC_Os08g02996 (4.6E−145)	Bradi1g28320 (3.5E−221)
V.UP	*Loc_Os04g59310 (5.3E*−*138)*	*Bradi5g27230 (7.0E*−*160)*	*S.UP*	*Loc_Os04g59310 (2.8E*−*137)*	*Bradi5g27230 (1.9E*−*159)*
V.Kin-2^b^	*Loc_Os04g59320 (1.5E*−*201)*	*Bradi5g27240 (2.4E*−*222)*	*S.Kin-3* ^*b*^	*Loc_Os04g59320 (1.5E*−*201)*	*Bradi5g27240 (2.4E*−*222)*

For abbreviations see the footnotes under Fig. 1

Italic boxes indicate the syntenic interval of *Rphq2* with rice chromosome 4 (indicated by Loc_Os**04**) and *Brachypodium* chromosome 5 (indicated by Bradi**5**)

*Na* not annotated, *x* no hit or no significant hit

^a^WBE114 is located within this annotated gene

^b^WBE115 is located within this annotated gene

## Discussion

### Two barley BAC libraries for the isolation of genes involved in basal resistance

Two non-gridded BAC libraries were constructed for Vada and SusPtrit barley genotypes. The average insert size over the complete Vada BAC library was 81 Kbp, with individual clones ranging from 18 to 209 Kbp (Supplemental Fig. 2a; Supplemental Table 2). The average insert size over the complete SusPtrit BAC library was 108 Kbp, with individual clones ranging from 33 to 274 Kbp (Supplemental Fig. 2b; Supplemental Table 4). This variation in insert sizes corresponds to the size selected fractions of DNA fragments ranging from 50 to 250 Kbp (i.e., fractions H1–H3). On average, SusPtrit inserts are 27 Kbp longer than Vada inserts. The average insert size of the Vada BAC library is the smallest among the BAC libraries available for barley. Such small average insert size is also observed in BAC libraries from other plant species such as wheat (Janda et al. 2004; Nilmalgoda et al. 2003) and soy bean (Xia et al. 2014). The average insert size of the Vada and SusPtrit BAC libraries is still comparable to three of the Morex BAC libraries (HVVMRXALLrA, HVVMRXALLhB and HVVMRXALLhC) recently made available by Schulte et al. (2011). However, the average insert size of the constructed BAC libraries is smaller than the one reported for the Cebada Capa BAC library (140 Kbp) which was constructed using the same protocol (Isidore et al. 2005). If a BAC library is to be used for genome-wide physical mapping and genome sequencing, then maximizing the average size of inserts is essential to limit walking. However, if a BAC library is to be used for positional cloning of genes that have already been confined to a very small interval, then having a large number of clones is more important to increase the chance to find the gene of interest. The principal aim of our BAC libraries is to isolate genes involved in basal resistance to cereal rust fungi after their high-resolution genetic mapping.

Based on the observed insert sizes of the BAC clones, the estimated genome coverage of the Vada BAC library is 2.6× and of the SusPtrit BAC library 3.7×. The genome coverage of the Vada library is comparable to the genome coverage of the Morex HVVMRXALLhB library, and SusPtrit to HVVMRXALLeA (Schulte et al. 2011). The estimation of the genome coverage based on microsatellite markers indicates, however, coverage of 5.0× and 6.8× for the Vada and the SusPtrit BAC libraries, respectively. The discrepancy between both estimations may be due to an underestimation of the average size of the BAC clones. As it is often observed in monocots (Peterson et al. 2000), several bands of identical sizes may be obtained after *Not*I restriction of BAC clones, which may result in the underestimation of the insert size from some clones. On the other hand, half of the microsatellite markers used for screening the BAC pools were derived from barley ESTs/genes (i.e., EST-SSR markers), implying that a marker amplifying a member from a gene family may in some cases amplify other genes from the same family as well unless the sequences of the primer pairs are unique (Thiel et al. 2003). Indeed, the pressure of a primer to anneal on a similar but not identical sequence is much stronger on BAC DNA than it is on full genomic DNA. Therefore, the genome coverage of the two libraries remains uncertain, but is probably slightly higher for SusPtrit than for Vada. Based on insert sizes, the BAC library of Vada gives at least 93 % probability of identifying a clone corresponding to any sequence of Vada and for BAC library of SusPtrit a probability of 98 % is expected (Clarke and Carbon 1976). Together, the two BAC libraries give more than 99 % probability of recovering any specific sequence from the barley genome.

### Haplotype divergence at the Rphq2 partial resistance locus

The *Rphq2* locus of Vada was donated by *H. laevigatum* (Arru et al. 2003; Giese et al. 1993). The name of the latter accession is taxonomically invalid, since it suggests a different (wild) species in the *Hordeum* genus. However, it is perfectly crossable with *H. vulgare* accessions and also has the *H. vulgare* general morphology, including non-shattering spikes. Therefore, it should be regarded as *H. vulgare.* It occurs in the ancestry of many West-European cultivars, including Emir, Delta and Minerva [Hickey et al. 2012; Germplasm Resources Information Network (GRIN) http://www.ars-grin.gov/npgs/holdings.html] and their derivatives. SusPtrit was bred from a double cross; Menelik/L100//Trigo Biasa/Nigrinudum (GRIN; Atienza et al. 2004). The very low degree (or even absence) of homology in the *Rphq2* region was supposed to be due to the West-European cultivar Vada and hence due to the donor line *H. laevigatum* that contributed this chromosome section (Marcel et al. 2007a). Other barley lines, viz the Ethiopian L94, the American cultivar Morex and SusPtrit as a descendant from various exotic barley accessions, seem to have maintained their homology in this area, as it appears from the alignment of the Morex genome sequences with the SusPtrit sequences and not with the Vada sequences (Table 4). Accordingly, the primer pairs designed on SusPtrit sequences did amplify DNA of L94 while primer pairs designed on Vada sequences did not. This lack of homology implies that having a BAC library from the parent donor of the resistance allele (Vada) is a necessity, and that if we would have relied only on the BAC library of Morex, we might have had great difficulties to acquire sequence information for Vada in that region and to identify the candidate genes for resistance.

Highly divergent sequences are usually implicated in suppression of recombination, as it was reported to occur commonly throughout the maize genome (Fu and Dooner 2002; Gore et al. 2009). Intraspecific haplotype divergence seems particularly common at resistance loci giving evidence for the dynamic evolution of those genome regions. Such haplotype divergence may arise from different mechanisms, including separate retrotransposon invasion between different genotypes of a same species (*Rph7* resistance to leaf rust in barley; Scherrer et al. 2005), gene duplication and unequal recombination in clustered multigene families (*RPP5* resistance to *Hyaloperonospora arabidopsidis* in Arabidopsis: Noël et al. 1999) or introgression of new resistance/susceptibility genes from landraces or wild-related species into modern cultivars (*Yr36* resistance to stripe rust in wheat: Fu et al. 2009; *Tsn1* resistance to tan spot disease of wheat: Faris et al. 2013). Such divergent sequences have been systemically associated with suppression of recombination in the target regions, hampering map-based cloning of the genes of interest. Recently, the genetic dissection of the *Sr2* adult plant stem rust resistance locus, introgressed from tetraploid emmer wheat into the modern hexaploid bread wheat cultivar Hope, revealed a major haplotype divergence including an insertion of a Germin-Like Protein (GLP) gene cluster in Hope (Mago et al. 2014). The GLP cluster represents a common presence–absence polymorphism in wheat but presence of the cluster does not correlate with the presence of *Sr2*, indicating that a GLP member of the cluster and not the cluster itself likely confers *Sr2* resistance. Suppressed recombination due to strong haplotype divergence and presence of a complex cluster of GLPs render the map-based cloning of *Sr2* a strenuous exercise (Mago et al. 2014).

Thus, a practical consequence of the strong haplotype divergence at the *Rphq2* locus is that we do not expect further recombination between V.Perox-2/S.Perox-2 and V.Kin-1/S.Kin-2, as in heterozygous material this chromosome region will probably hardly pair. Indeed we failed to obtain recombinant plants in this region after screening more than 3000 plants (data not shown). The lack of recombination is also suggested by the repulsion between V.Perox-3 (Vada specific) and S.UF + S.Kin-1 (SusPtrit specific) in a set of 194 barley accessions, including some Ethiopian, Indonesian, East-Asian, American, European and *H. spontaneum* accessions. We labeled these haplotypes PAA (presence, absence absence) when they only amplified the V.Perox-3 sequence, and APP when they only amplified the S.UF and S.Kin-1 sequences. Among the 194 tested barley accessions, no other haplotype than the PAA and APP haplotypes were detected, suggesting an early and complete split in the haplotypes and a strict repression of recombination in this chromosome region. Suppression of recombination prevents further fine mapping of *Rphq2* using the Vada and SusPtrit or Vada and L94 heterozygous material. Nevertheless, among the 111 accessions having the PAA haplotype, we observed variation from a high level of partial resistance (including Vada and *H. laevigatum*) to a high level of susceptibility to barley leaf rust (data not shown). Susceptible PAA accessions are likely to carry a susceptible *rphq2* allele and to recombine with the *Rphq2* locus in Vada, allowing further fine mapping of *Rphq2*.

### Multiple candidate genes at the *Rphq2* locus

The annotation of sequences at *Rphq2* using TriAnnot_v3.5 with the specific barley default analysis identified 12 genes in Vada and 12 genes in SusPtrit, but only five of the annotated genes are shared between Vada and SusPtrit. Out of the five annotated genes in common between Vada and SusPtrit, four were also conserved in rice and *B. distachyon*. No nucleotide-binding site-leucine-rich repeat (NBS-LRR) resistance gene was annotated in the *Rphq2* region. A study of differentially expressed genes between L94 and L94-*Rphq2* NIL suggested seven candidates for *Rphq2* (Chen et al. 2010). Among the candidates, only one gene, unigene2111 (encoding a peroxidase), was similar to an annotated gene at *Rphq2*. Unigene2111 has 99 % identity with the coding sequence of V.Perox-2 of Vada and S.Perox-2 of SusPtrit. This suggests peroxidase-2 as a good candidate to explain *Rphq2*. This is also supported by the fact that peroxidases are known to be involved in defense reactions, i.e., cell wall reinforcement and hypersensitive reaction [reviewed in Hückelhoven and Kogel (2003); Almagro et al. (2009)]. Furthermore, González et al. (2010) found 61 % of the QTLs for partial resistance to adapted rust fungi (including *Rphq2*) to co-localize with peroxidase-based markers. The kinases are also good candidates to explain *Rphq2*. Kinases are involved in various signaling pathways including plant defense system against pathogens [Reviewed in Rodriguez et al. (2010); Antolín-Llovera et al. (2012)]. The annotated peroxidase and kinase genes co-segregating with *Rphq2* resistance phenotype have all been found to be expressed in leaf tissue after leaf rust infection, making them good candidate genes to explain *Rphq2*. It is possible that one of the peroxidase or kinase genes identified in the physical window of *Rphq2* affects the resistance phenotype observed. Another possibility is that peroxidase or kinase gene members function as a complex QTL as observed in rice where the resistance effect of a QTL on chromosome 8 was shown to be contributed by a cluster of germin-like protein genes (Manosalva et al. 2009).

A gene from the SINA protein family was annotated only on the *Rphq2* sequence of Vada. Based on its function, this might be a candidate gene for *Rphq2* as well. SINA proteins are E3 ligases with a RING finger domain at the N-terminal followed by a conserved SINA domain which has a function in substrate binding and dimerization (Hu and Fearon 1999). One particular SINA protein is found, in a symbiotic interaction, to impair the rhizobial infection in *Medicago truncatula* (Mbengue et al. 2010) and *Lotus japonicus* (Den Herder et al. 2012). However, no support of expression was found for V-SINA in our RNA-seq data, making it a less likely candidate gene to explain *Rphq2*. The candidate genes for *Rphq2* resemble none of the partial resistance genes cloned previously (Fu et al. 2009; Fukuoka et al. 2009; Hayashi et al. 2010; Krattinger et al. 2009; Manosalva et al. 2009). Genes for partial resistance can be resistance factors, such as ABC transporter gene of *Lr34* (Krattinger et al. 2009), or susceptibility factors, such as a proline-rich protein of Pi21 (Fukuoka et al. 2009). Therefore, the candidate genes of *Rphq2* might be either a resistance factor from Vada or a susceptibility factor from SusPtrit.

Stable transformation of candidate genes for resistance in a susceptible barley genotype can be performed using Golden SusPtrit, a new genetically well-transformable barley line (Yeo et al. 2014). Golden SusPtrit inherited the susceptibility of SusPtrit to *P. hordei* and to non-adapted rust fungi, as well as the transformability of Golden Promise. The transformants in the genetic background of Golden SusPtrit will allow the testing of *Rphq2* candidate genes affecting resistance to adapted and non-adapted rust fungi. This will be valuable information to understand partial resistance in barley and its possible association with non-host resistance.

#### **Author contribution statement**

FYKS: did much of the experimental work; wrote the first draft, and implemented all suggestions by co-authors, YW: advised on the annotation and did the haplotyping of 194 accessions. TV: developed molecular markers. CH: assisted TCM in developing the BAC libraries. PL: did the sequences annotation using Triannot pipeline. BC: supervised TCM in developing the BAC library. XQQ: supervised the transcriptomics and annotation studies. REN: overall supervision, and editing of the various drafts and submission. TCM: designed the study, developed the BAC libraries, and actively supervised FYKS in writing.

## Electronic supplementary material

Supplementary material 1 (DOCX 293 kb)

## References

[CR2] Allouis S, Moore G, Bellec A, Sharp R, Rampant PF, Mortimer K, Pateyron S, Foote TN, Griffiths S, Caboche M (2003). Construction and characterisation of a hexaploid wheat (*Triticum aestivum* L.) BAC library from the reference germplasm’Chinese Spring’. Cereal Res Commun.

[CR3] Almagro L, Gómez Ros LV, Belchi-Navarro S, Bru R, Ros Barceló A, Pedreño MA (2009). Class III peroxidases in plant defence reactions. J Exp Bot.

[CR4] Antolín-Llovera M, Ried MK, Binder A, Parniske M (2012). Receptor kinase signaling pathways in plant-microbe interactions. Annu Rev Phytopathol.

[CR5] Arru L, Francia E, Pecchioni N (2003). Isolate-specific QTLs of resistance to leaf stripe (*Pyrenophora graminea*) in the ‘Steptoe’ × ’Morex’ spring barley cross. Theor Appl Genet.

[CR6] Atienza S, Jafary H, Niks RE (2004). Accumulation of genes for susceptibility to rust fungi for which barley is nearly a nonhost results in two barley lines with extreme multiple susceptibility. Planta.

[CR7] Brugmans B, van der Hulst RGM, Visser RGF, Lindhout P, van Eck HJ (2003). A new and versatile method for the successful conversion of AFLP™ markers into simple single locus markers. Nucleic Acids Res.

[CR8] Brugmans B, Hutten RGB, Rookmaker ANO, Visser RGF, Eck HJ (2006). Exploitation of a marker dense linkage map of potato for positional cloning of a wart disease resistance gene. Theor Appl Genet.

[CR9] Chalhoub B, Belcram H, Caboche M (2004). Efficient cloning of plant genomes into bacterial artificial chromosome (BAC) libraries with larger and more uniform insert size. Plant Biotechnol J.

[CR10] Chen X, Niks R, Hedley P, Morris J, Druka A, Marcel T, Vels A, Waugh R (2010). Differential gene expression in nearly isogenic lines with QTL for partial resistance to *Puccinia hordei* in barley. BMC Genom.

[CR11] Clarke L, Carbon J (1976). A colony bank containing synthetic CoI EI hybrid plasmids representative of the entire *E. coli* genome. Cell.

[CR12] Den Herder G, Yoshida S, Antolín-Llovera M, Ried MK, Parniske M (2012). *Lotus japonicus* E3 ligase SEVEN IN ABSENTIA4 destabilizes the symbiosis receptor-like kinase SYMRK and negatively regulates rhizobial infection. Plant Cell.

[CR13] Dros J (1957). The creation and maintenance of two spring barley varieties. Euphytica.

[CR14] Faris JD, Liu Z, Xu SS (2013). Genetics of tan spot resistance in wheat. Theor Appl Genet.

[CR15] Fu H, Dooner HK (2002). Intraspecific violation of genetic colinearity and its implications in maize. Proc Natl Acad Sci USA.

[CR16] Fu D, Uauy C, Distelfeld A, Blechl A, Epstein L, Chen X, Sela H, Fahima T, Dubcovsky J (2009). A kinase-START gene confers temperature-dependent resistance to wheat stripe rust. Science.

[CR17] Fukuoka S, Saka N, Koga H, Ono K, Shimizu T, Ebana K, Hayashi N, Takahashi A, Hirochika H, Okuno K, Yano M (2009). Loss of function of a proline-containing protein confers durable disease resistance in rice. Science.

[CR18] Giese H, Holm-Jensen AG, Jensen HP, Jensen J (1993). Localization of the *Laevigatum* powdery mildew resistance gene to barley chromosome 2 by the use of RFLP markers. Theor Appl Genet.

[CR19] González AM, Marcel TC, Kohutova Z, Stam P, van der Linden CG, Niks RE (2010). Peroxidase profiling reveals genetic linkage between peroxidase gene clusters and basal host and non-host resistance to rusts and mildew in barley. PLoS One.

[CR20] Gore MA, Chia J, Elshire RJ, Sun Q, Ersoz ES, Hurwitz BL, Peiffer JA, McMullen MD, Grills GS, Ross-Ibarra J, Ware DH, Buckler ES (2009). A first-generation haplotype map of maize. Science.

[CR21] Hayashi N, Inoue H, Kato T, Funao T, Shirota M, Shimizu T, Kanamori H, Yamane H, Hayano-Saito Y, Matsumoto T, Yano M, Takatsuji H (2010). Durable panicle blast-resistance gene Pb1 encodes an atypical CC-NBS-LRR protein and was generated by acquiring a promoter through local genome duplication. Plant J.

[CR22] Hickey LT, Lawson W, Platz GJ, Dieters M, Franckowiak J (2012). Origin of leaf rust adult plant resistance gene Rph20 in barley. Genome.

[CR23] Hu G, Fearon ER (1999). Siah-1 N-terminal RING domain is required for proteolysis function, and C-terminal sequences regulate oligomerization and binding to target proteins. Mol Cell Biol.

[CR24] Hückelhoven R, Kogel K-H (2003). Reactive oxygen intermediates in plant-microbe interactions: who is who in powdery mildew resistance?. Planta.

[CR25] Isidore E, Scherrer B, Bellec A, Budin K, Faivre P, Waugh R, Keller B, Caboche M, Feuillet C, Chalhoub B (2005). Direct targeting and rapid isolation of BAC clones spanning a defined chromosome region. Funct Integr Genom.

[CR26] Jafary H, Szabo LJ, Niks RE (2006). Innate nonhost immunity in barley to different heterologous rust fungi is controlled by sets of resistance genes with different and overlapping specificities. Mol Plant Microbe Interact.

[CR27] Jafary H, Albertazzi G, Marcel TC, Niks RE (2008). High diversity of genes for nonhost resistance of barley to heterologous rust fungi. Genetics.

[CR28] Janda J, Bartoš J, Šafář J, Kubaláková M, Valárik M, Číhalíková J, Šimková H, Caboche M, Sourdille P, Bernard M, Chalhoub B, Doležel J (2004). Construction of a subgenomic BAC library specific for chromosomes 1D, 4D and 6D of hexaploid wheat. Theor Appl Genet.

[CR29] Johnston PA, Niks RE, Meiyalaghan V, Blanchet E, Pickering R (2013). Rph22: mapping of a novel leaf rust resistance gene introgressed from the non-host *Hordeum bulbosum* L. into cultivated barley (*Hordeum vulgare* L.). Theor Appl Genet.

[CR30] Jones JDG, Dangl JL (2006). The plant immune system. Nature.

[CR31] Kraakman ATW, Martínez F, Mussiraliev B, Eeuwijk FA, Niks RE (2006). Linkage disequilibrium mapping of morphological, resistance, and other agronomically relevant traits in modern spring barley cultivars. Mol Breed.

[CR32] Krattinger SG, Lagudah ES, Spielmeyer W, Singh RP, Huerta-Espino J, McFadden H, Bossolini E, Selter LL, Keller B (2009). A putative ABC transporter confers durable resistance to multiple fungal pathogens in wheat. Science.

[CR33] Kurtz S, Phillippy A, Delcher A, Smoot M, Shumway M, Antonescu C, Salzberg S (2004). Versatile and open software for comparing large genomes. Genome Biol.

[CR34] Leroy P, Guilhot N, Sakai H, Bernard A, Choulet F, Theil S, Reboux S, Amano N, Flutre T, Pelegrin C, Ohyanagi H, Seidel M, Giacomoni F, Reichstadt M, Alaux M, Gicquello E, Legeai F, Cerutti L, Numa H, Tanaka T, Mayer K, Itoh T, Quesneville H, Feuillet C (2012). TriAnnot: a versatile and high performance pipeline for the automated annotation of plant genomes. Front Plant Sci.

[CR35] Ma Z, Weining S, Sharp PJ, Liu C (2000). Non-gridded library: a new approach for BAC (bacterial artificial chromosome) exploitation in hexaploid wheat (*Triticum aestivum*). Nucleic Acids Res.

[CR36] Mago R, Tabe L, Vautrin S, Šimková H, Kubaláková M, Upadhyaya N, Berges H, Kong X, Breen J, Doležel J, Appels R, Ellis JG, Spielmeyer W (2014). Major haplotype divergence including multiple germin-like protein genes, at the wheat *Sr2* adult plant stem rust resistance locus. BMC Plant Biol.

[CR37] Manosalva PM, Davidson RM, Liu B, Zhu X, Hulbert SH, Leung H, Leach JE (2009). A germin-like protein gene family functions as a complex quantitative trait locus conferring broad-spectrum disease resistance in rice. Plant Physiol.

[CR38] Marcel TC, Aghnoum R, Durand J, Varshney RK, Niks RE (2007). Dissection of the barley 2L1. 0 region carrying the ‘*Laevigatum*’ quantitative resistance gene to leaf rust using near-isogenic lines (NIL) and subNIL. Mol Plant Microbe Interact.

[CR39] Marcel TC, Varshney RK, Barbieri M, Jafary H, de Kock MJD, Graner A, Niks RE (2007). A high-density consensus map of barley to compare the distribution of QTLs for partial resistance to *Puccinia hordei* and of defence gene homologues. Theor Appl Genet.

[CR40] Marcel TC, Gorguet B, Ta MT, Kohutova Z, Vels A, Niks RE (2008). Isolate specificity of quantitative trait loci for partial resistance of barley to *Puccinia hordei* confirmed in mapping populations and near-isogenic lines. New Phytol.

[CR41] Mayer KFX, Martis M, Hedley PE, Šimková H, Liu H, Morris JA, Steuernagel B, Taudien S, Roessner S, Gundlach H, Kubaláková M, Suchánková P, Murat F, Felder M, Nussbaumer T, Graner A, Salse J, Endo T, Sakai H, Tanaka T, Itoh T, Sato K, Platzer M, Matsumoto T, Scholz U, Doležel J, Waugh R, Stein N (2011). Unlocking the barley genome by chromosomal and comparative genomics. Plant Cell.

[CR42] Mbengue M, Camut S, de Carvalho-Niebel F, Deslandes L, Froidure S, Klaus-Heisen D, Moreau S, Rivas S, Timmers T, Hervé C, Cullimore J, Lefebvre B (2010). The *Medicago truncatula* E3 ubiquitin ligase PUB1 interacts with the LYK3 symbiotic receptor and negatively regulates infection and nodulation. Plant Cell.

[CR43] Meyers BC, Kaushik S, Nandety RS (2005). Evolving disease resistance genes. Curr Opin Plant Biol.

[CR44] Niks RE (1983). Comparative histology of partial resistance and the nonhost reaction to leaf rust pathogens in barley and wheat seedlings. Phytopathology.

[CR45] Niks RE (1983). Haustorium formation by Puccinia hordei in leaves of hypersensitive, partially resistant, and nonhost plant genotypes. Phytopathology.

[CR46] Niks RE (1987). Nonhost plant species as donors for resistance to pathogens with narrow host range I. Determination of nonhost status. Euphytica.

[CR47] Niks RE, Marcel TC (2009). Nonhost and basal resistance: how to explain specificity?. New Phytol.

[CR48] Niks RE, Fernandez E, Bv Haperen, Bekele Aleye B, Martinez F (2000). Specificity of QTLs for partial and non-host resistance of barley to leaf rust fungi. Acta Phytopathol Entomol Hun.

[CR49] Niks RE, Parlevliet JE, Lindhout P, Bai Y (2011) Breeding crops with resistance to diseases and pests. Wageningen Academic Publishers, Wageningen, p 198

[CR50] Nilmalgoda SD, Cloutier S, Walichnowski AZ (2003). Construction and characterization of a bacterial artificial chromosome (BAC) library of hexaploid wheat (*Triticum aestivum* L.) and validation of genome coverage using locus-specific primers. Genome.

[CR51] Noël L, Moores TL, van der Biezen EA, Parniske M, Daniels MJ, Parker JE, Jones JDG (1999). Pronounced intraspecific haplotype divergence at the *RPP5* complex disease resistance locus of Arabidopsis. Plant Cell.

[CR52] Parlevliet JE, van Ommeren A (1975). Partial resistance of barley to leaf rust, *Puccinia hordei*. II. Relationship between field trials, micro plot tests and latent period. Euphytica.

[CR53] Peterson DG, Tomkins JP, Frisch DA, Wing RA, Paterson AH (2000). Construction of plant bacterial artificial chromosome (BAC) libraries: an illustrated guide. J Agric Genom.

[CR54] Qi X, Niks RE, Stam P, Lindhout P (1998). Identification of QTLs for partial resistance to leaf rust (*Puccinia hordei*) in barley. Theor Appl Genet.

[CR55] Qi X, Jiang G, Chen W, Niks RE, Stam P, Lindhout P (1999). Isolate-specific QTLs for partial resistance to *Puccinia hordei* in barley. Theor Appl Genet.

[CR56] Rodriguez MCS, Petersen M, Mundy J (2010). Mitogen-activated protein kinase signaling in plants. Annu Rev Plant Biol.

[CR57] Saisho D, Myoraku E, Kawasaki S, Sato K, Takeda K (2007). Construction and characterization of a bacterial artificial chromosome (BAC) library from the Japanese malting barley variety ‘Haruna Nijo’. Breed Sci.

[CR58] Sakata K, Nagamura Y, Numa H, Antonio BA, Nagasaki H, Idonuma A, Watanabe W, Shimizu Y, Horiuchi I, Matsumoto T, Sasaki T, Higo K (2002). RiceGAAS: an automated annotation system and database for rice genome sequence. Nucleic Acids Res.

[CR59] Salvaudon L, Giraud T, Shykoff JA (2008). Genetic diversity in natural populations: a fundamental component of plant–microbe interactions. Curr Opin Plant Biol.

[CR60] Sambrook J, Fritsch EF, Maniatis T (1989) Molecular cloning, a laboratory manual, 2nd edn. Cold Spring Harbor Laboratory Press, Cold Spring Harbor, p 626

[CR61] Scherrer B, Isidore E, Klein P, Kim J-S, Bellec A, Chalhoub B, Keller B, Feuillet C (2005). Large intraspecific haplotype variability at the *Rph7* locus results from rapid and recent divergence in the barley genome. Plant Cell.

[CR62] Schulte D, Ariyadasa R, Shi B, Fleury D, Saski C, Atkins M, Wu C, Graner A, Langridge P, Stein N (2011). BAC library resources for map-based cloning and physical map construction in barley (*Hordeum vulgare* L.). BMC Genom.

[CR63] Shen J, Araki H, Chen L, Chen J, Tian D (2006). Unique evolutionary mechanism in R-genes under the presence/absence polymorphism in *Arabidopsis thaliana*. Genetics.

[CR64] Shi BJ, Sutton T, Collins NC, Pallotta M, Langridge P (2010). Construction of a barley bacterial artificial chromosome library suitable for cloning genes for boron tolerance, sodium exclusion and high grain zinc content. Plant Breed.

[CR65] The International Barley Genome Sequencing Consortium (2012). A physical, genetic and functional sequence assembly of the barley genome. Nature.

[CR66] Thiel T, Michalek W, Varshney R, Graner A (2003). Exploiting EST databases for the development and characterization of gene-derived SSR-markers in barley (*Hordeum vulgare* L.). Theor Appl Genet.

[CR67] Trujillo M, Troeger M, Niks RE, Kogel K, Hückelhoven R (2004). Mechanistic and genetic overlap of barley host and non-host resistance to *Blumeria graminis*. Mol Plant Pathol.

[CR68] van Berloo R, Aalbers H, Werkman A, Niks R (2001). Resistance QTL confirmed through development of QTL-NILs for barley leaf rust resistance. Mol Breed.

[CR69] Varshney R, Marcel TC, Ramsay L, Russell J, Röder MS, Stein N, Waugh R, Langridge P, Niks RE, Graner A (2007). A high density barley microsatellite consensus map with 775 SSR loci. Theor Appl Genet.

[CR70] Xia Z, Wu H, Watanabe S, Harada K (2014). Construction and targeted retrieval of specific clone from a non-gridded soybean bacterial artificial chromosome library. Anal Biochem.

[CR71] Yeo FKS, Hensel G, Vozábová T, Martin-Sanz A, Marcel TC, Kumlehn J, Niks RE (2014). Golden SusPtrit: a genetically well transformable barley line for studies on the resistance to rust fungi. Theor Appl Genet.

[CR72] Yu Y, Tomkins JP, Waugh R, Frisch DA, Kudrna D, Kleinhofs A, Brueggeman RS, Muehlbauer GJ, Wise RP, Wing RA (2000). A bacterial artificial chromosome library for barley (*Hordeum vulgare* L.) and the identification of clones containing putative resistance genes. Theor Appl Genet.

