# Supplementary material for: Haplotype divergence and multiple candidate genes at Rphq2, a partial resistance QTL of barley to Puccinia hordei
Source: Theor Appl Genet. 2015 Nov 5;129:289–304. doi: 10.1007/s00122-015-2627-5 (PMC4733143; doi:10.1007/s00122-015-2627-5)
Supplement: Supplementary file 1 — Supplementary material 1 (DOCX 293 kb) [file 122_2015_2627_MOESM1_ESM.docx]

**List of Supplemental Material**

**Supplemental Material 1** Detailed methodology for the construction and characterization of the pooled BAC libraries

**Supplemental Material 1** Detailed methodology for the construction and characterization of the pooled BAC libraries

The methodology followed to construct the two BAC libraries has been described in detail by Peterson et al. (2000), with several modifications proposed in subsequent reports (Allouis et al. 2003; Chalhoub et al. 2004; Isidore et al. 2005).

*Preparation of high-molecular-weight DNA*

About 500 seeds from Vada and from SusPtrit were sown in plastic trays and placed in a greenhouse compartment. Leaves were harvested twice from the same plants between two and four-weeks after sowing, flash-frozen in liquid nitrogen and stored at -80 ^°^C. Before each harvest the plants were kept in the dark during 48 hours to promote the burst of chloroplasts and limit chloroplast DNA in the BAC libraries. About 40-50 grams of leaf were used to extract nuclei and prepare high molecular weight (HMW) DNA following the protocol described by Peterson et al. (2000), with modifications (Allouis et al. 2003; Chalhoub et al. 2004). The modifications consisted mainly in the omission of polyvinyl pyrolidone 40000 (PVP-40) and ascorbic acid from the sucrose-based extraction buffer (SEB) and from the lysis buffer. The quantity of PVP-40 in the wash buffers (WB-A, -B, -C) was lowered to 0.25% instead of 2%.

*Partial digestion and size fractionation*

Plugs of HMW DNA were prepared in 0.75% InCert® agarose (BMA) as described by Peterson et al. (2000). Twelve plugs macerated in the *Hind*III modified restriction (H3M) buffer were digested at different enzyme concentrations: 0.2, 0.5, 1.0, 2.0, 5.0, 7.5, 10.0, 15.0, 20.0 and 40.0 units. The partial digestion was performed in a 37 ^°^C water bath during exactly 20 min. Then, the plugs were migrated together on a 1.0% SeaKem® Gold agarose gel (Cambrex) in 0.25x TBE in a CHEF-Mapper apparatus (Bio-Rad) with the following conditions: pulse linear ramping from 1 to 40 sec, angle 120^°^, current 6.0 V/cm and 21 hours run time at 14 ^°^C. After electrophoresis, the partially digested DNA was subjected to a single size selection. The flanking lanes loaded with the lambda ladder PFGE marker (New England Biolabs) were removed from the gel and stained with ethidium bromide to indicate the location of the size ranges. For each library, five slices of agarose-containing DNA in the ranges 50-100 kb (H0 fraction), 100-150 kb (H1 fraction), 150-200 kb (H2 fraction), 200-250 kb (H3 fraction) and 250-300 (H4 fraction) were excised from the gel and stored at 4 ^°^C in 1x TAE buffer.

The HMW DNA was isolated by electro-elution using a BioRad Electroelution system run one hour at 60 mA direct current and 90 V alternating current. From each agarose slice, 40 to 80 μl was recovered with a wide-bored tip.

*Ligation and transformation*

The insert DNA from H0 to H4 fractions was ligated separately into the pIndigoBAC vector (CalTech) prepared for high efficiency cloning with *Hind*III as described by Chalhoub et al. (2004) or into the commercial pIndigoBAC-5 vector (Epicentre Biotechnologies). Ligations were performed in a 50 μl reaction volume with 33 μl insert DNA (50-100 ng), 50 ng of vector DNA, 10 μl of 5x reaction buffer and 5 units of T4 DNA ligase (Invitrogen). Ligation mixtures were incubated at 16 ^°^C overnight and dialyzed 90 min at 4 ^°^C as described by Chalhoub et al. (2004). Sixteen microliters of de-salted ligation were mixed with 110 μl ElectroMax DH10B electrocompetent cells (Invitrogen). Seventeen microliters of the mixture were electroporated at 330 V and the electroporations were pooled in a tube containing 2 ml SOC medium (Sambrook et al. 1989) with 0.3 ml of 2 M glucose.

*Pooling of the BAC clones*

Transformed cells diluted with SOC were incubated at 37 ^°^C under gentle agitation (220 rpm) for 60 min and plated on a selective LB medium (Luria-Bertani medium) with 12.5 μg chloramphenicol (CAM), 0.55 mM IPTG (isopropylthio-β-D-galactoside) and 80 μg/ml X-Gal (5-bromo-4-chloro-3-indolyl-β-D-galactoside) (Sambrook et al. 1989). A test plating of each transformation was performed in order to allow an average of about 1,500 colonies per plate as suggested by Isidore et al. (2005). The plates were incubated at 37 ^°^C for 20 hours. The clones were collected from each plate in 3 ml storage buffer (LB medium supplemented with 50% glycerol) and homogenized for 30 min under gentle agitation (220 rpm). Each of the resulting 3 ml cultures represents a pool. The pools were then aliquot into four tubes, each corresponding to one copy of the library (copies A, B, C and D). Copy A is stored in a -80 ^°^C freezer at Unité de Recherche en Génomique Végétale (URGV – Evry, France), copy B at Institute of Botany, Chinese Academy of Sciences (IBCAS – Beijing, China). Copies C and D are stored at Wageningen UR, Plant Breeding (WUR – the Netherlands).

*Characterization of the BAC libraries*

Twenty-four BAC clones were randomly selected from the fractions H1, H2 and H3 of each library (i.e. 72 BAC clones per library) and grown for 24 hours at 37 ^°^C in 1.5 ml LB medium containing 12.5 μg CAM. The BAC DNA was extracted following an alkaline lysis procedure (Sambrook et al. 1989) with ready-to-use buffers P1, P2 and P3 (Qiagen) and digested overnight with *Not*I (New England Biolabs). Digested products were separated on a 1% SeaKem® LE agarose gel (BMA) in 0.5x TBE in a CHEF-DR™ II apparatus (BioRad) with the following pulsed field gel electrophoresis parameters: 200 V, 5-15 sec switch time, for 14.3 hours at 10 ^°^C. The insert sizes of selected BAC clones were estimated after comparison with the CHEF DNA size standard lambda ladder (Bio-Rad) run in the same gel.

BAC-pool DNA was isolated from 250 μl aliquot per pool from the copy D of the libraries as described previously. The two barley BAC libraries were characterized for genome representation by PCR-screening of 46 pools per library with one microsatellite marker from each of the 14 barley chromosome arms. The markers were selected from the barley microsatellite consensus map of Varshney et al. (2007). The reverse primer of each microsatellite was labeled with IRDye-700 or IRDye-800 and the PCR-product visualized on a LICOR 4200 DNA sequencer (LICOR® Biosciences).

*Screening the library for BAC clones spanning the* Rphq2 *locus*

The work flow for identifying Vada and SusPtrit BAC clones spanning the *Rphq2* locus is presented in Fig. 1. The solid and liquid selective LB media (here onwards LBA and LB, respectively) used were supplemented with 20 μg/ml of CAM unless indicated otherwise. The BAC libraries were screened following a PCR based method (Fig. 1a). In a first step, we used molecular markers known to be closely linked to *Rphq2* to screen the BAC pools and to identify positive BAC clones. In a second step, after the identification and sequencing of several positive BAC clones, we used primers designed to amplify the BAC-end-sequences (bes), the genes annotated in these clones and newly developed markers. We also used primers to amplify sequences at the edges of gaps in the already assembled BAC sequences (Table 1).

For each BAC pool that was positive in the PCR screen, test plating was performed in order to allow an average of about 2,000 to 3000 colonies on a large square Petri dish (506.25 cm^2^) which is used for robotic picking of BAC clones (Fig. 1b). Each positive BAC pool was then diluted accordingly and 2 ml of the diluted positive BAC pools were plated on large Petri dishes containing LBA (Fig. 1c). The BAC clones were grown at 37 ^°^C for about 14 hours, and afterwards kept at 4 ^°^C until use. The BAC clones on the large Petri dishes were picked using a picking robot (Genomic Solutions Flexsys picker) and cultured in 384-well plates containing LB-freeze [LB, 36 mM K_2_HPO_4_, 13.2 mM KH_2_PO_4_, 1.7 mM trisodium citrate, 0.4 mM MgSO_4_, 6.8 mM (NH_4_)_2_SO_4_, 4.4% (vol/vol) glycerol] with 20 μg/ml CAM. The picked BAC clones were grown at 37 ^°^C for 14 to 16 hours and stored at -80 ^°^C (Fig. 1d). Each 384-well plate was replicated onto a small square Petri dish (144 cm^2^) containing LBA. The replicates were kept at 37 ^°^C overnight. On each plate, 3 ml of LB were added and the clones were scraped and collected (sub-pool) into a culture tube (Fig. 1e). The DNA of each sub-pool was isolated (Sambrook et al. 1989) and PCR-screened to identify the positive sub-pool. The 384-well plate corresponding to the positive sub-pool was replicated again as described above onto three small square Petri dishes (Fig. 1f).

In one of the small square Petri dishes, BAC clones from each column were pooled (24 column-pools). In another Petri dish, BAC clones from each row were pooled (16 row-pools) (Fig. 1g). The pooling was done by streaking across a column or row using an autoclaved toothpick and the pooled BAC clones were cultured overnight at 37 ^°^C in culture tube containing 3 ml of LB. The DNA of each column- and row-pool was isolated (Sambrook et al. 1989) and PCR-screened to identify positive pools. The intersection between a positive column-pool and row-pool indicates a positive BAC clone. The positive BAC clone was picked from the third Petri dish using a toothpick and grown in a culture tube containing 3 ml LB at 37 ^°^C overnight. The liquid culture was then diluted 10,000 to 20,000x and 100 μl of the culture was plated on a small round Petri dish (ø 94 x 16 mm) containing LBA and grown at 37 ^°^C overnight. Five single-colonies were picked using a toothpick and grown as described above (labeled as ‘a’ to ‘e’). Their DNA was isolated (Sambrook et al. 1989) and the colonies validated after PCR-amplification with the markers and primers used during the screening process. The positive single-colonies were maintained in glycerol stock (LB supplemented with 25% glycerol). The insert size of the BAC clones was determined as described in the previous section before further analyses (see below). The BAC clones were named as follow: “BAC pool number; Sub-pool number (384-well plate number); row letter; column number; single-colony letter” (eg. V41 P7 L 3 A).

*BAC clones fingerprinting*

All the confirmed positive BAC clones from the Vada and SusPtrit libraries were fingerprinted following the AFLP procedure from Brugmans et al. (2006) using the H*ind*III/*Taq*I restriction enzyme combination. The generated fragments were separated on a LICOR 4200 DNA sequencer (LI-COR Biosciences, Lincoln, NE, USA). The fingerprints were scored manually. Shared bands between BAC clones indicated sequence overlaps between the clones.

*BAC-end sequencing*

The extremities of all the confirmed positive BAC clones were sequenced (i.e. BAC-ends sequencing). The clones were digested individually with 12 different blunt-end restriction endonucleases (*Alu*I, *Bsh*1236I, *BspL*I, *Bsu*RI, *Dpn*I, *Dra*I, *Exo*32I, *Hin*cII, *KspA*I, *Rsa*I, *Sca*I, *Sma*I and *Ssp*I), and ligated with non-specific blunt adapters (genome walker adapter). The restriction-ligations were performed in 50 μl reaction volume comprising 50-100 ng DNA template, 1x restriction and ligation buffer (RL buffer), 0.02 unit T4 DNA ligase, 0.1 unit restriction enzyme, 0.5 μM genome walker adapter (GWadp; top: 5’-GTAATACGACTCACT ATAGGGCACGCGTGGTCGACGGCCCGGGCTGGA-3’; bottom: 5’-PO_4_-TCCAGCCC-NH_2_-3’), and 0.2 mM adenosine triphosphate (ATP). The reactions were incubated overnight at 37 °C and diluted 20x with MilliQ water (RL-DNA). For each sample, a nested PCR approach with two rounds of amplification were carried out on the 12 RL-DNA in order to obtain PCR products as specific as possible before sequencing. The first PCR was performed in 20 μl reaction volume comprising 5 μl RL-DNA, 1x PCR buffer, 0.2 mM dNTPs, 0.15 mM forward-1 pIndigoBAC/ pIndigoBAC-5 primer (5’-GGATGTGCTGCAAGGCGATTAAGTTGG-3’), 0.15 mM adapter primer-1 (5’-TAATACGACTCACTATAGGGC-3’), and 0.02 unit Taq DNA polymerase. A separate PCR reaction was performed using the reverse-1 pIndigoBAC/ pIndigoBAC-5 primer (5’-CTCGTATGTTGTGTGGAATTGTGAGC-3’). The first-PCR product was diluted 50x in MilliQ water (Merck Millipore) and 5 μl of the diluted PCR product was used as template for the second PCR. The second PCR was performed in 20 μl reaction volume. The PCR reaction mixture was similar to the first PCR, except for the primers that were used. They were the forward-2 pIndigoBAC/pIndigoBAC-5 primer (5’-ACGGCCAGTGAATTGTAATA-3’) and adapter primer-2 (5’-ACTATAGGGCACGCGTGGT-3’). A separate PCR reaction was performed using the reverse-2 pIndigoBAC/pIndigoBAC-5 primer (5’-GGAAACAGCTATGAC CATGA-3’).

The first PCR reaction was carried out with 24 cycles of 30 seconds denaturation at 94 °C, 30 seconds annealing at 56 °C and 90 seconds extension at 72 °C (Profile A). The second PCR reaction was carried out with 5 min of initial denaturation at 94 °C, 35 cycles of Profile A and 7 min of final extension at 72 °C. The final PCR product was visualized on 1% agarose gel. For each BAC clone, the largest fragment (depending on the initial restriction endonuclease) obtained in the final PCR product of the forward and of the reverse PCR reactions was selected for sequencing. Primers which amplify the BAC-ends were designed by using Lasergene software (DNASTAR® 8 Inc., Madison, WI, USA).

**List of Supplemental Figures**

**Supplemental Fig. 1** Work flow for the identification of BAC clones spanning the Rphq2 locus in Vada and in SusPtrit.

**Supplemental Fig. 2** Analysis of 24 randomly selected barley BAC clones from (a) Vada, fraction VH2, and (b) SusPtrit, fraction SH3, on an ethidium bromide-stained CHEF gel (5-15 sec switch time, 14.3 hours) showing insert DNA above and below the common 7.5 Kbp pIndigoBAC or pIndigoBAC-5 vector band.

**Supplemental Fig. 1** Work flow for the identification of BAC clones spanning the Rphq2 locus in Vada and in SusPtrit.

|   (a) |   (b) |
| --- | --- |
| **Supplemental Fig. 2** Analysis of 24 randomly selected barley BAC clones from (a) Vada, fraction VH2, and (b) SusPtrit, fraction SH3, on an ethidium bromide-stained CHEF gel (5-15 sec switch time, 14.3 hours) showing insert DNA above and below the common 7.5 Kbp pIndigoBAC or pIndigoBAC-5 vector band. | |

**List of Supplemental Tables**

| **Supplemental Table 1** The descriptions of the 116 BAC pools of Vada.  **Supplemental Table 2** Composition of the Vada BAC library |
| --- |
| **Supplemental Table 3** The descriptions of the 110 BAC pools of SusPtrit.  **Supplemental Table 4** Composition of the SusPtrit BAC library  **Supplemental Table 5** PCR-based screening of the Vada and SusPtrit BAC libraries with microsatellite markers representing each chromosome arm of barley on a subset of 46 pools per library (representing approximately 1.4 and 1.9 barley genome-equivalents) |
| **Supplemental Table 6** The positive BAC pools from Vada and SusPtrit BAC libraries detected using 12 PCR primers. |
| **Supplemental Table 7**  The primers used for determining the order of BAC clones in Table 3. |
| **Supplemental Table 8** The 41 molecular markers developed and mapped between flanking markers WBE114 and WBE115 of *Rphq2*. |
| **Supplemental Table 9** Haplotypes of 194 barley accessions based on the presence (P) and absence (A) of V.Perox-3, S.UF and S.Kin-1. |
|  |

| **Supplemental Table 1** The descriptions of the 116 BAC pools of Vada. | | | | | | |
| --- | --- | --- | --- | --- | --- | --- |
| Pool | White ^a^ | Blue ^a^ | Fraction | Vector | Insert size | Individual pool genome contribution ^b^ |
| V1 | 20 | 0 | H0 | pIndigoBAC-5 | 83 | 1660 |
| V2 | 411 | 0 | H0 | pIndigoBAC-5 | 83 | 34113 |
| V3 | 244 | 0 | H0 | pIndigoBAC-5 | 83 | 20252 |
| V4 | 864 | 30 | H1 | pIndigoBAC | 99 | 85536 |
| V5 | 1871 | 39 | H1 | pIndigoBAC | 99 | 185229 |
| V6 | 1871 | 39 | H1 | pIndigoBAC | 99 | 185229 |
| V7 | 1871 | 39 | H1 | pIndigoBAC | 99 | 185229 |
| V8 | 1871 | 39 | H1 | pIndigoBAC | 99 | 185229 |
| V9 | 1871 | 39 | H1 | pIndigoBAC | 99 | 185229 |
| V10 | 1871 | 39 | H1 | pIndigoBAC | 99 | 185229 |
| V11 | 1871 | 39 | H1 | pIndigoBAC | 99 | 185229 |
| V12 | 966 | 12 | H1 | pIndigoBAC-5 | 83 | 80178 |
| V13 | 156 | 4 | H1 | pIndigoBAC | 111 | 17316 |
| V14 | 2604 | 76 | H1 | pIndigoBAC | 111 | 289044 |
| V15 | 2604 | 76 | H1 | pIndigoBAC | 111 | 289044 |
| V16 | 2604 | 76 | H1 | pIndigoBAC | 111 | 289044 |
| V17 | 2604 | 76 | H1 | pIndigoBAC | 111 | 289044 |
| V18 | 2604 | 76 | H1 | pIndigoBAC | 111 | 289044 |
| V19 | 2604 | 76 | H1 | pIndigoBAC | 111 | 289044 |
| V20 | 2604 | 76 | H1 | pIndigoBAC | 111 | 289044 |
| V21 | 1160 | 50 | H1 | pIndigoBAC-5 | 83 | 96280 |
| V22 | 966 | 12 | H1 | pIndigoBAC-5 | 83 | 80178 |
| V23 | 1871 | 39 | H1 | pIndigoBAC | 83 | 155293 |
| V24 | 966 | 12 | H1 | pIndigoBAC-5 | 83 | 80178 |
| V25 | 966 | 12 | H1 | pIndigoBAC-5 | 83 | 80178 |
| V26 | 966 | 12 | H1 | pIndigoBAC-5 | 83 | 80178 |
| V27 | 966 | 12 | H1 | pIndigoBAC-5 | 83 | 80178 |
| V28 | 966 | 12 | H1 | pIndigoBAC-5 | 83 | 80178 |
| V29 | 966 | 12 | H1 | pIndigoBAC-5 | 83 | 80178 |
| V30 | 966 | 12 | H1 | pIndigoBAC-5 | 83 | 80178 |
| V31 | 966 | 12 | H1 | pIndigoBAC-5 | 83 | 80178 |
| V32 | 606 | 12 | H1 | pIndigoBAC-5 | 96 | 58176 |
| V33 | 1786 | 67 | H1 | pIndigoBAC-5 | 96 | 171456 |
| V34 | 1786 | 67 | H1 | pIndigoBAC-5 | 96 | 171456 |
| V35 | 1786 | 67 | H1 | pIndigoBAC-5 | 96 | 171456 |
| V36 | 1786 | 67 | H1 | pIndigoBAC-5 | 96 | 171456 |

| **Supplemental Table 1** Cont… | | | | | | |
| --- | --- | --- | --- | --- | --- | --- |
| Pool | White ^a^ | Blue ^a^ | Fraction | Vector | Insert size | Individual pool genome contribution ^b^ |
| V37 | 1786 | 67 | H1 | pIndigoBAC-5 | 96 | 171456 |
| V38 | 1786 | 67 | H1 | pIndigoBAC-5 | 96 | 171456 |
| V39 | 1786 | 67 | H1 | pIndigoBAC-5 | 96 | 171456 |
| V40 | 1786 | 67 | H1 | pIndigoBAC-5 | 96 | 171456 |
| V41 | 1786 | 67 | H1 | pIndigoBAC-5 | 96 | 171456 |
| V42 | 1786 | 67 | H1 | pIndigoBAC-5 | 96 | 171456 |
| V43 | 1786 | 67 | H1 | pIndigoBAC-5 | 96 | 171456 |
| V44 | 1786 | 67 | H1 | pIndigoBAC-5 | 96 | 171456 |
| V45 | 319 | 1 | H2 | pIndigoBAC | 94 | 29986 |
| V46 | 2136 | 34 | H2 | pIndigoBAC | 94 | 200784 |
| V47 | 2136 | 34 | H2 | pIndigoBAC | 94 | 200784 |
| V48 | 2136 | 34 | H2 | pIndigoBAC | 94 | 200784 |
| V49 | 2136 | 34 | H2 | pIndigoBAC | 94 | 200784 |
| V50 | 2136 | 34 | H2 | pIndigoBAC | 94 | 200784 |
| V51 | 2136 | 34 | H2 | pIndigoBAC | 94 | 200784 |
| V52 | 84 | 0 | H2 | pIndigoBAC | 129 | 10836 |
| V53 | 995 | 8 | H2 | pIndigoBAC | 129 | 128355 |
| V54 | 995 | 8 | H2 | pIndigoBAC | 129 | 128355 |
| V55 | 995 | 8 | H2 | pIndigoBAC | 129 | 128355 |
| V56 | 995 | 8 | H2 | pIndigoBAC | 129 | 128355 |
| V57 | 995 | 8 | H2 | pIndigoBAC | 129 | 128355 |
| V58 | 725 | 31 | H2 | pIndigoBAC-5 | 55 | 39875 |
| V59 | 1947 | 57 | H2 | pIndigoBAC-5 | 55 | 107085 |
| V60 | 1947 | 57 | H2 | pIndigoBAC-5 | 55 | 107085 |
| V61 | 1947 | 57 | H2 | pIndigoBAC-5 | 55 | 107085 |
| V62 | 1947 | 57 | H2 | pIndigoBAC-5 | 55 | 107085 |
| V63 | 1947 | 57 | H2 | pIndigoBAC-5 | 55 | 107085 |
| V64 | 1947 | 57 | H2 | pIndigoBAC-5 | 55 | 107085 |
| V65 | 1947 | 57 | H2 | pIndigoBAC-5 | 55 | 107085 |
| V66 | 508 | 12 | H2 | pIndigoBAC-5 | 50 | 25400 |
| V67 | 2051 | 178 | H2 | pIndigoBAC-5 | 50 | 102550 |
| V68 | 2149 | 144 | H2 | pIndigoBAC-5 | 50 | 107450 |
| V69 | 2100 | 161 | H2 | pIndigoBAC-5 | 50 | 105000 |
| V70 | 2100 | 161 | H2 | pIndigoBAC-5 | 50 | 105000 |
| V71 | 2100 | 161 | H2 | pIndigoBAC-5 | 50 | 105000 |
| V72 | 2100 | 161 | H2 | pIndigoBAC-5 | 50 | 105000 |

| **Supplemental Table 1** Cont… | | | | | | |
| --- | --- | --- | --- | --- | --- | --- |
| Pool | White ^a^ | Blue ^a^ | Fraction | Vector | Insert size | Individual pool genome contribution ^b^ |
| V73 | 2100 | 161 | H2 | pIndigoBAC-5 | 50 | 105000 |
| V74 | 2100 | 161 | H2 | pIndigoBAC-5 | 50 | 105000 |
| V75 | 2100 | 161 | H2 | pIndigoBAC-5 | 50 | 105000 |
| V76 | 2100 | 161 | H2 | pIndigoBAC-5 | 50 | 105000 |
| V77 | 2100 | 161 | H2 | pIndigoBAC-5 | 50 | 105000 |
| V78 | 2100 | 161 | H2 | pIndigoBAC-5 | 50 | 105000 |
| V79 | 2100 | 161 | H2 | pIndigoBAC-5 | 50 | 105000 |
| V80 | 168 | 5 | H3 | pIndigoBAC | 104 | 17472 |
| V81 | 377 | 11 | H3 | pIndigoBAC | 104 | 39208 |
| V82 | 377 | 11 | H3 | pIndigoBAC | 104 | 39208 |
| V83 | 377 | 11 | H3 | pIndigoBAC | 104 | 39208 |
| V84 | 377 | 11 | H3 | pIndigoBAC | 104 | 39208 |
| V85 | 46 | 3 | H3 | pIndigoBAC | 40 | 1840 |
| V86 | 515 | 8 | H3 | pIndigoBAC | 40 | 20600 |
| V87 | 515 | 8 | H3 | pIndigoBAC | 40 | 20600 |
| V88 | 1322 | 34 | H3 | pIndigoBAC-5 | 92 | 121624 |
| V89 | 1024 | 5 | H3 | pIndigoBAC-5 | 92 | 94208 |
| V90 | 1024 | 5 | H3 | pIndigoBAC-5 | 92 | 94208 |
| V91 | 1024 | 5 | H3 | pIndigoBAC-5 | 92 | 94208 |
| V92 | 1024 | 5 | H3 | pIndigoBAC-5 | 92 | 94208 |
| V93 | 1024 | 5 | H3 | pIndigoBAC-5 | 92 | 94208 |
| V94 | 1024 | 5 | H3 | pIndigoBAC-5 | 92 | 94208 |
| V95 | 1024 | 5 | H3 | pIndigoBAC-5 | 92 | 94208 |
| V96 | 1024 | 5 | H3 | pIndigoBAC-5 | 92 | 94208 |
| V97 | 1024 | 5 | H3 | pIndigoBAC-5 | 92 | 94208 |
| V98 | 1024 | 5 | H3 | pIndigoBAC-5 | 92 | 94208 |
| V99 | 1024 | 5 | H3 | pIndigoBAC-5 | 92 | 94208 |
| V100 | 1024 | 5 | H3 | pIndigoBAC-5 | 92 | 94208 |
| V101 | 870 | 10 | H3 | pIndigoBAC-5 | 54 | 46980 |

| **Supplemental Table 1** Cont… | | | | | | |
| --- | --- | --- | --- | --- | --- | --- |
| Pool | White ^a^ | Blue ^a^ | Fraction | Vector | Insert size | Individual pool genome contribution ^b^ |
| V102 | 1294 | 42 | H3 | pIndigoBAC-5 | 54 | 69876 |
| V103 | 1294 | 42 | H3 | pIndigoBAC-5 | 54 | 69876 |
| V104 | 1294 | 42 | H3 | pIndigoBAC-5 | 54 | 69876 |
| V105 | 1294 | 42 | H3 | pIndigoBAC-5 | 54 | 69876 |
| V106 | 1294 | 42 | H3 | pIndigoBAC-5 | 54 | 69876 |
| V107 | 1294 | 42 | H3 | pIndigoBAC-5 | 54 | 69876 |
| V108 | 1294 | 42 | H3 | pIndigoBAC-5 | 54 | 69876 |
| V109 | 1294 | 42 | H3 | pIndigoBAC-5 | 54 | 69876 |
| V110 | 1294 | 42 | H3 | pIndigoBAC-5 | 54 | 69876 |
| V111 | 1294 | 42 | H3 | pIndigoBAC-5 | 54 | 69876 |
| V112 | 1294 | 42 | H3 | pIndigoBAC-5 | 54 | 69876 |
| V113 | 1294 | 42 | H3 | pIndigoBAC-5 | 54 | 69876 |
| V114 | 20 | 1 | H4 | pIndigoBAC-5 | 83 | 1660 |
| V115 | 112 | 0 | H4 | pIndigoBAC-5 | 83 | 9296 |
| V116 | 158 | 0 | H4 | pIndigoBAC-5 | 83 | 13114 |
| ^a^ The estimated number of white and blue colonies.  ^b^ The genome contribution of the individual pool calculated by multiplying the number of white colonies with the  estimated insert size. | | | | | | |

| **Supplemental Table 2** Composition of the Vada BAC library | | | | | |
| --- | --- | --- | --- | --- | --- |
| Size selection range  (Kbp) | Number of pools | White colonies ^a^ | Blue colonies ^a^ | Insert size ^b^  (Kbp) | Coverage ^c^  (Mbp) |
| H0: 50-100 | 3 | 225 | 0 | - ^d^ | - |
| H1: 100-150 | 41 | 1636 | 45 | 98.3 | 6,593.6 |
| H2: 150-200 | 35 | 1724 | 79 | 67.4 | 4,066.9 |
| H3: 200-250 | 34 | 963 | 20 | 71.9 | 2,354.1 |
| H4: 250-300 | 3 | 97 | 1 | - ^d^ | - |
| **Total** | **116** | **1389** | **46** | **81.2** | **13,014.6** |
| ^a^ Estimation of the average number of colonies per pool  ^b^ Average insert size estimated with 24 randomly selected BAC clones per fraction  ^c^ Calculated with the estimated number of white colonies and their average insert size  ^d^ Because of their low number, the clones from fractions H0 and H4 have been neglected | | | | | |

| **Supplemental Table 3** The descriptions of the 110 BAC pools of SusPtrit. | | | | | | |
| --- | --- | --- | --- | --- | --- | --- |
| Pool | White ^a^ | Blue ^a^ | Fraction | Vector | Insert size | Individual pool genome contribution ^b^ |
| S1 | 218 | 11 | H0 | pIndigoBAC-5 | 89 | 19402 |
| S2 | 3703 | 2 | H0 | pIndigoBAC-5 | 89 | 329567 |
| S3 | 3703 | 2 | H0 | pIndigoBAC-5 | 89 | 329567 |
| S4 | 3703 | 2 | H0 | pIndigoBAC-5 | 89 | 329567 |
| S5 | 3703 | 2 | H0 | pIndigoBAC-5 | 89 | 329567 |
| S6 | 3703 | 2 | H0 | pIndigoBAC-5 | 89 | 329567 |
| S7 | 3703 | 2 | H0 | pIndigoBAC-5 | 89 | 329567 |
| S8 | 3703 | 2 | H0 | pIndigoBAC-5 | 89 | 329567 |
| S9 | 3703 | 2 | H0 | pIndigoBAC-5 | 89 | 329567 |
| S10 | 1957 | 40 | H1 | pIndigoBAC | 95 | 185915 |
| S11 | 790 | 14 | H1 | pIndigoBAC | 95 | 75050 |
| S12 | 1957 | 40 | H1 | pIndigoBAC | 95 | 185915 |
| S13 | 1957 | 40 | H1 | pIndigoBAC | 95 | 185915 |
| S14 | 1957 | 40 | H1 | pIndigoBAC | 95 | 185915 |
| S15 | 1957 | 40 | H1 | pIndigoBAC | 95 | 185915 |
| S16 | 1957 | 40 | H1 | pIndigoBAC | 95 | 185915 |
| S17 | 1957 | 40 | H1 | pIndigoBAC | 95 | 185915 |
| S18 | 276 | 0 | H1 | pIndigoBAC | 124 | 34224 |
| S19 | 2609 | 61 | H1 | pIndigoBAC | 124 | 323516 |
| S20 | 2609 | 61 | H1 | pIndigoBAC | 124 | 323516 |
| S21 | 2609 | 61 | H1 | pIndigoBAC | 124 | 323516 |
| S22 | 2609 | 61 | H1 | pIndigoBAC | 124 | 323516 |
| S23 | 2609 | 61 | H1 | pIndigoBAC | 124 | 323516 |
| S24 | 1702 | 86 | H1 | pIndigoBAC-5 | 106 | 180412 |
| S25 | 1126 | 23 | H1 | pIndigoBAC-5 | 106 | 119356 |
| S26 | 1126 | 23 | H1 | pIndigoBAC-5 | 106 | 119356 |
| S27 | 1126 | 23 | H1 | pIndigoBAC-5 | 106 | 119356 |
| S28 | 1126 | 23 | H1 | pIndigoBAC-5 | 106 | 119356 |
| S29 | 1126 | 23 | H1 | pIndigoBAC-5 | 106 | 119356 |
| S30 | 1126 | 23 | H1 | pIndigoBAC-5 | 106 | 119356 |
| S31 | 1126 | 23 | H1 | pIndigoBAC-5 | 106 | 119356 |
| S32 | 1126 | 23 | H1 | pIndigoBAC-5 | 106 | 119356 |
| S33 | 1126 | 23 | H1 | pIndigoBAC-5 | 106 | 119356 |
| S34 | 1126 | 23 | H1 | pIndigoBAC-5 | 106 | 119356 |
| S35 | 1126 | 23 | H1 | pIndigoBAC-5 | 106 | 119356 |
| S36 | 1126 | 23 | H1 | pIndigoBAC-5 | 106 | 119356 |

| **Supplemental Table 3** Cont… | | | | | | |
| --- | --- | --- | --- | --- | --- | --- |
| Pool | White ^a^ | Blue ^a^ | Fraction | Vector | Insert size | Individual pool genome contribution ^b^ |
| S37 | 1126 | 23 | H1 | pIndigoBAC-5 | 106 | 119356 |
| S38 | 1126 | 23 | H1 | pIndigoBAC-5 | 106 | 119356 |
| S39 | 578 | 4 | H1 | pIndigoBAC-5 | 104 | 60112 |
| S40 | 2522 | 82 | H1 | pIndigoBAC-5 | 104 | 262288 |
| S41 | 2522 | 82 | H1 | pIndigoBAC-5 | 104 | 262288 |
| S42 | 2522 | 82 | H1 | pIndigoBAC-5 | 104 | 262288 |
| S43 | 2522 | 82 | H1 | pIndigoBAC-5 | 104 | 262288 |
| S44 | 2522 | 82 | H1 | pIndigoBAC-5 | 104 | 262288 |
| S45 | 2522 | 82 | H1 | pIndigoBAC-5 | 104 | 262288 |
| S46 | 2522 | 82 | H1 | pIndigoBAC-5 | 104 | 262288 |
| S47 | 280 | 9 | H2 | pIndigoBAC | 108 | 30240 |
| S48 | 1954 | 32 | H2 | pIndigoBAC | 108 | 211032 |
| S49 | 1954 | 32 | H2 | pIndigoBAC | 108 | 211032 |
| S50 | 1954 | 32 | H2 | pIndigoBAC | 108 | 211032 |
| S51 | 1954 | 32 | H2 | pIndigoBAC | 108 | 211032 |
| S52 | 1954 | 32 | H2 | pIndigoBAC | 108 | 211032 |
| S53 | 27 | 0 | H2 | pIndigoBAC | 161 | 4347 |
| S54 | 609 | 6 | H2 | pIndigoBAC | 161 | 98049 |
| S55 | 609 | 6 | H2 | pIndigoBAC | 161 | 98049 |
| S56 | 609 | 6 | H2 | pIndigoBAC | 161 | 98049 |
| S57 | 609 | 6 | H2 | pIndigoBAC | 161 | 98049 |
| S58 | 609 | 6 | H2 | pIndigoBAC | 161 | 98049 |
| S59 | 1464 | 54 | H2 | pIndigoBAC-5 | 116 | 169824 |
| S60 | 1029 | 28 | H2 | pIndigoBAC-5 | 116 | 119364 |
| S61 | 1029 | 28 | H2 | pIndigoBAC-5 | 116 | 119364 |
| S62 | 1029 | 28 | H2 | pIndigoBAC-5 | 116 | 119364 |
| S63 | 1029 | 28 | H2 | pIndigoBAC-5 | 116 | 119364 |
| S64 | 1029 | 28 | H2 | pIndigoBAC-5 | 116 | 119364 |
| S65 | 1029 | 28 | H2 | pIndigoBAC-5 | 116 | 119364 |
| S66 | 1029 | 28 | H2 | pIndigoBAC-5 | 116 | 119364 |
| S67 | 1029 | 28 | H2 | pIndigoBAC-5 | 116 | 119364 |
| S68 | 1029 | 28 | H2 | pIndigoBAC-5 | 116 | 119364 |
| S69 | 1029 | 28 | H2 | pIndigoBAC-5 | 116 | 119364 |
| S70 | 1029 | 28 | H2 | pIndigoBAC-5 | 116 | 119364 |
| S71 | 1029 | 28 | H2 | pIndigoBAC-5 | 116 | 119364 |
| S72 | 460 | 2 | H2 | pIndigoBAC-5 | 105 | 48300 |

| **Supplemental Table 3** Cont... | | | | | | |
| --- | --- | --- | --- | --- | --- | --- |
| Pool | White ^a^ | Blue ^a^ | Fraction | Vector | Insert size | Individual pool genome contribution ^b^ |
| S73 | 3113 | 102 | H2 | pIndigoBAC-5 | 105 | 326865 |
| S74 | 3113 | 102 | H2 | pIndigoBAC-5 | 105 | 326865 |
| S75 | 3113 | 102 | H2 | pIndigoBAC-5 | 105 | 326865 |
| S76 | 3113 | 102 | H2 | pIndigoBAC-5 | 105 | 326865 |
| S77 | 3113 | 102 | H2 | pIndigoBAC-5 | 105 | 326865 |
| S78 | 3113 | 102 | H2 | pIndigoBAC-5 | 105 | 326865 |
| S79 | 3113 | 102 | H2 | pIndigoBAC-5 | 105 | 326865 |
| S80 | 3113 | 102 | H2 | pIndigoBAC-5 | 105 | 326865 |
| S81 | 3113 | 102 | H2 | pIndigoBAC-5 | 105 | 326865 |
| S82 | 3113 | 102 | H2 | pIndigoBAC-5 | 105 | 326865 |
| S83 | 3113 | 102 | H2 | pIndigoBAC-5 | 105 | 326865 |
| S84 | 3113 | 102 | H2 | pIndigoBAC-5 | 105 | 326865 |
| S85 | 209 | 5 | H3 | pIndigoBAC | 154 | 32186 |
| S86 | 949 | 9 | H3 | pIndigoBAC | 154 | 146146 |
| S87 | 949 | 9 | H3 | pIndigoBAC | 154 | 146146 |
| S88 | 949 | 9 | H3 | pIndigoBAC | 154 | 146146 |
| S89 | 949 | 9 | H3 | pIndigoBAC | 154 | 146146 |
| S90 | 949 | 9 | H3 | pIndigoBAC | 154 | 146146 |
| S91 | 949 | 9 | H3 | pIndigoBAC | 154 | 146146 |
| S92 | 25 | 0 | H3 | pIndigoBAC | 168 | 4200 |
| S93 | 199 | 1 | H3 | pIndigoBAC | 168 | 33432 |
| S94 | 303 | 0 | H3 | pIndigoBAC | 168 | 50904 |
| S95 | 251 | 1 | H3 | pIndigoBAC | 168 | 42168 |
| S96 | 251 | 1 | H3 | pIndigoBAC | 168 | 42168 |
| S97 | 251 | 1 | H3 | pIndigoBAC | 168 | 42168 |
| S98 | 346 | 13 | H3 | pIndigoBAC-5 | 124 | 42904 |
| S99 | 491 | 21 | H3 | pIndigoBAC-5 | 124 | 60884 |
| S100 | 491 | 21 | H3 | pIndigoBAC-5 | 124 | 60884 |
| S101 | 491 | 21 | H3 | pIndigoBAC-5 | 124 | 60884 |

| **Supplemental Table 3** Cont... | | | | | | |
| --- | --- | --- | --- | --- | --- | --- |
| Pool | White ^a^ | Blue ^a^ | Fraction | Vector | Insert size | Individual pool genome contribution ^b^ |
| S102 | 491 | 21 | H3 | pIndigoBAC-5 | 124 | 60884 |
| S103 | 491 | 21 | H3 | pIndigoBAC-5 | 124 | 60884 |
| S104 | 491 | 21 | H3 | pIndigoBAC-5 | 124 | 60884 |
| S105 | 128 | 4 | H3 | pIndigoBAC-5 | 127 | 16256 |
| S106 | 849 | 24 | H3 | pIndigoBAC-5 | 127 | 107823 |
| S107 | 849 | 24 | H3 | pIndigoBAC-5 | 127 | 107823 |
| S108 | 849 | 24 | H3 | pIndigoBAC-5 | 127 | 107823 |
| S109 | 849 | 24 | H3 | pIndigoBAC-5 | 127 | 107823 |
| S110 | 849 | 24 | H3 | pIndigoBAC-5 | 127 | 107823 |
| ^a^ The estimated number of white and blue colonies  ^b^ The genome contribution of the individual pool calculated by multiplying the number of white colonies with the  estimated insert size | | | | | | |

| **Supplemental Table 4** Composition of the SusPtrit BAC library | | | | | |
| --- | --- | --- | --- | --- | --- |
| Size selection range  (Kbp) | Number of pools | White colonies ^a^ | Blue colonies ^a^ | Insert size ^b^  (Kbp) | Coverage ^c^  (Mbp) |
| H0: 50-100 | 9 | 3,316 | 3 | 83.0 ^d^ | 2,477.1 |
| H1: 100-150 | 37 | 1,716 | 43 | 106.7 | 6,775.8 |
| H2: 150-200 | 38 | 1,704 | 48 | 110.5 | 7,152.9 |
| H3: 200-250 | 26 | 571 | 12 | 140.6 | 2,087.7 |
| H4: 250-300 | - | - | - | - | - |
| **Total** | **110** | **1,572** | **34** | **108.0** | **18,493.5** |
| ^a^ Estimation of the average number of colonies per pool  ^b^ Average insert size estimated with 24 randomly selected BAC clones per fraction  ^c^ Calculated with the estimated number of white colonies and their average insert size  ^d^ Corresponds to the insert size of a single BAC clone isolated from fraction H0 | | | | | |

| **Supplemental Table 5** PCR-based screening of the Vada and SusPtrit BAC libraries with microsatellite markers representing each chromosome arm of barley on a subset of 46 pools per library (representing approximately 1.4 and 1.9 barley genome-equivalents) | | | | | | |
| --- | --- | --- | --- | --- | --- | --- |
| Chrom. | Position  (cM) ^a^ | Microsatellite | Vada allele ^b^  (bp) | SusPtrit allele ^b^  (bp) | Nr. Pools  Vada ^c^ | Nr. Pools  SusPtrit ^c^ |
| 1HS | 25.0 | GBMS062 | 127 | 127 | 2 | 8 |
| 1HL | 73.4 | Bmac0032 | - | 220 | 1 | 2 |
| 2HS | 28.6 | HVM36 | 126 | 108 | 6 | 4 |
| 2HL | 90.9 | GBM1062 | 215 | 219 | 3 | 2 |
| 3HS | 35.4 | scssr10559 | 210 | 216 | 3 | 4 |
| 3HL | 150.5 | HVM62 | 260 | 250 | 2 | 4 |
| 4HS | 47.1 | GBM1482 | 210 | 210 | 12 | 5 |
| 4HL | 125.5 | GBM1015 | 232 | 220 | 0 | 1 |
| 5HS | 21.2 | GBM1176 | - | 300 | 1 | 4 |
| 5HL | 88.0 | Bmag0223 | 174 | 170 | 1 | 5 |
| 6HS | 4.6 | Bmac0316 | 169 | 165 | 1 | 3 |
| 6HL | 129.2 | GBM1087 | 201 | 198 | 4 | 2 |
| 7HS | 18.6 | Bmag0007 | 200 | 204 | 2 | 4 |
| 7HL | 87.7 | GBM1359 | 151 | 145 | 0 | 1 |
| ^a^ Position of the corresponding microsatellite marker on the barley integrated map, Marcel 2009  available at http://wheat.pw.usda.gov/GG2/index.shtml (Aghnoum et al. 2010)  ^b^ Approximate size of the allele amplified on Vada or SusPtrit genomic DNA  ^c^ Number of positive pools per library | | | | | | |

| **Supplemental Table 6** The positive BAC pools from Vada and SusPtrit BAC libraries detected using 12 PCR primers. | | | | | | | | | | | | | |
| --- | --- | --- | --- | --- | --- | --- | --- | --- | --- | --- | --- | --- | --- |
|  | WBE114 | P15M51-204 | besV76P5D5AR | S35P100001F4 | S35P100004F2 | besS35P2K14EF | *Rphq2*.S01 | S7300002F | *Rphq2*.V30 | *Rphq2*.V32 | P14M54-252 | | WBE115 |
| Vada BAC library | V11 |  |  |  | NT |  |  |  | NT | NT |  | |  |
|  |  | V17 |  |  | NT |  |  |  | NT | NT |  | |  |
|  | V21 |  |  |  | NT |  |  |  | NT | NT |  | |  |
|  |  |  | V35 |  | NT |  |  |  | NT | NT |  | |  |
|  | V38 |  |  |  | NT |  |  |  | NT | NT |  | |  |
|  | V41 |  |  |  | NT |  |  |  | NT | NT |  | | V41 |
|  | V45 |  |  |  | NT |  |  |  | NT | NT |  | |  |
|  | V48 |  | V48 |  | NT |  |  |  | NT | NT |  | | V48 |
|  |  | V51 |  |  | NT |  |  |  | NT | NT |  | |  |
|  |  | V69 |  |  | NT |  |  |  | NT | NT |  | |  |
|  |  | V76 | V76 |  | NT |  |  |  | NT | NT |  | |  |
|  |  | V77 |  |  | NT |  |  |  | NT | NT |  | |  |
|  | V84 |  |  |  | NT |  |  |  | NT | NT |  | |  |
|  | V89 |  |  |  | NT |  |  |  | NT | NT |  | |  |
|  |  |  |  |  | NT |  |  |  | NT | NT |  | | V104 |
|  | V113 |  |  |  | NT |  |  |  | NT | NT |  | |  |
| SusPtrit BAC library | S7 |  |  |  |  | S7 | S7 |  |  |  | S7 | | S7 |
|  | S8 |  |  |  |  |  |  |  |  |  |  | |  |
|  |  |  |  |  |  |  | S13 |  |  |  | S13 | |  |
|  | S22 |  |  |  |  |  |  |  |  |  |  | |  |
|  | S23 |  |  |  |  |  |  |  |  |  |  | |  |
|  | S30 |  |  |  |  |  |  |  |  |  |  | |  |
|  | S35 |  |  | S35 | S35 | S35 | S35 |  | S35 |  | S35 | | S35 |
|  |  |  |  |  |  |  |  |  |  | S36 |  | |  |
|  | S39 |  |  |  |  |  |  |  |  |  |  | |  |
|  | S40 |  |  | S40 | S40 | S40 |  |  |  | S40 |  | |  |
|  | S43 |  |  |  |  |  |  |  |  |  |  | | S43 |
|  | S45 |  |  | S45 | S45 |  | S45 | S45 |  |  | S45 | |  |
|  |  |  |  |  |  | S51 | S51 |  |  |  | S51 | | S51 |
|  | S56 |  |  |  |  |  |  |  |  |  |  | |  |
|  |  |  |  |  |  |  |  |  | S58 |  | S58 | |  |
|  |  |  |  |  |  |  | S73 |  | S73 |  | S73 | |  |
|  | S75 |  |  | S75 |  |  |  |  |  | S75 |  | |  |
|  |  |  |  |  |  |  |  |  | S77 |  |  | |  |
|  |  |  |  |  |  |  |  |  | S81 |  |  | | S81 |
|  | S82 |  |  |  |  |  |  |  |  |  |  | |  |
|  |  |  |  |  |  |  | S109 |  | S109 |  | S109 | | S109 |
| The shaded areas indicate no amplification was expected from the primers on the respective BAC library.  NT; Not tested | | | | | | | | | | | |  |  |

| **Supplemental Table 7**  The primers used for determining the order of BAC clones in Table 6. | | | |
| --- | --- | --- | --- |
| Name | Primers sequences (5’- 3’) | Ta (°C) | Source |
| WBE114 | Refer Table 1 |  | Marcel et al. (2007a) |
| WBE115 | Refer Table 1 |  | Marcel et al. (2007a) |
| besV41P7L3AF | *F:* GTTGCTTCATGTATACTTCTTCTT | 56 | BAC end |
|  | *R:* ATCTTCCCAACGTCAACAAATC |  |  |
| besV41P7L3AR | *F:* ATAATCTTAGCCCTCACATCACCA | 56 | BAC end |
|  | *R:* AGTTCCAAGCAAAGCGTCGTAG |  |  |
| besV76P5D5AF | *F:* ATAGGGATGCTTACCACTGAA | 56 | BAC end |
|  | *R:* AAATTACTAGCTAGACTCCCACTC |  |  |
| besV76P5D5AR | **Supplemental Table 8** |  | BAC end |
| besV48P5B18AR | **Supplemental Table 8** |  | BAC end |
| besV48P5B18AF | *F:* TACTATCCTTCCGCTCACAACTCA | 58 | BAC end |
|  | *R:* GGGACCCCTATTACCACCAG |  |  |
| *Rphq2*.S01 | **Supplemental Table 8** |  | SusPtrit annotated gene |
| P14M54-252 | **Supplemental Table 8** |  | Marcel et al. (2007a) |
| besS35P1J10AF | *F:* CTGCCACTCTTTATCTTTTTG | 56 | BAC end |
|  | *R:* TAGTATCGGGGAGTATTAGC |  |  |
| besS35P1J10AR | *F:* TCCGGTATGCACGAAAAC | 58 | BAC end |
|  | *R:* CCTGCCGGTAAACGAGAT |  |  |
| besS35P2K14EF | **Supplemental Table 8** |  | BAC end |
| besS35P2K14ER | *F:* TGTTCCGTTCATACTCACCTT | 56 | BAC end |
|  | *R:* ACCATAGAACGACCCTCACA |  |  |
| besS7P2C21EF | *F:* GAAGTATATGCCGACAACCAAATG | 58 | BAC end |
|  | *R:* GAAAACCGTCCAACCTCTACAAGT |  |  |
| besS81P2C6AF | *F:* CCCTAGGGAAAGCCATCATACG | 58 | BAC end |
|  | *R:* GGGTTTGCCTCATCCATAGC |  |  |
| besS81P2C6AR | *F:* CGCCGTTTTGACATCCATCTG | 58 | BAC end |
|  | *R:* TCAAATCCGAGGGCAAAGTGTT |  |  |
| bfsS35P2K14EF-267 | **Supplemental Table 8** |  | BAC AFLP |
| bfsS35P2K14EF-283 | *F:* ATGCGACCTATTGCATGTCT | 56 | BAC AFLP |
|  | *R:* TGACGGTAAACAAGCCTTTC |  |  |
| bfsS35P2K14EF-468 | *F:* CTCATGGAAGCAGCAAAACTA | 58 | BAC AFLP |
|  | *R:* GCCGGCATACTCACCACT |  |  |
|  |  |  |  |

**Supplemental Table 7 Reference**

Marcel, T. C., R. Aghnoum, J. Durand, R. K. Varshney and R. E. Niks, 2007a Dissection of the barley 2L1. 0 region carrying the '*Laevigatum*' quantitative resistance gene to leaf rust using near-isogenic lines (NIL) and subNIL. Mol. Plant-Microbe Interact. **20:** 1604-1615.

| **Supplemental Table 8** The 41 molecular markers developed and mapped between flanking markers WBE114 and WBE115 of *Rphq2*. | | | | |
| --- | --- | --- | --- | --- |
| Name | Tm  (°C) | Restriction  Enzyme | Primers sequences  (5’- 3’) | Source |
| Dominant markers amplifying Vada | | | | |
| besV76P5D5AR | 56 |  | *F:* GAGGAGCCGTGTCGTCTTGT | BAC end |
|  |  |  | *R:* CCGTTTCCGTTCACTGGTTAT |  |
| bfsS35P2K14E-267 | 56 |  | *F:* CGCCGTATACCAAGGCTATT | BAC AFLP |
|  |  |  | *R:* ATGAGCTCGTAGACCAGCAG |  |
| FQ2D1F6 | 65 |  | *F:* ATGTGGGCCAACGGTGCAAATCAGG | Vada BAC sequence |
|  |  |  | *R:* CAATACGGAGGTGTCGCCCATAAC |  |
| FQ2D3F | 65 |  | *F:* CGTCTGCGGCCCCGTCGTCTCC | Vada BAC sequence |
|  |  |  | *R:* GATGGGCGCGGTGGTCTTGTTCTTG |  |
| FQ2D4F9 | 65 |  | *F:* GCCCCGTGCATCCGTTCGT | Vada BAC sequence |
|  |  |  | *R:* TCCGCAGATTTCATAGGCAGGTGT |  |
| FQ2D4F14 | 65 |  | *F:* TTTGATGCGCAGGGTTTGGAGAGGT | Vada BAC sequence |
|  |  |  | *R:* GGGAGGGGTGAGGGGGCTGGAG |  |
| FQ2D4F15 | 65 |  | *F:* TCCTCCACGGCACCTACCAAGACG | Vada BAC sequence |
|  |  |  | *R:* CCCGGACGGACGCCTGAAG |  |
| FQ2D6F | 65 |  | *F:* TAGGGGCGATAGAACCAGAAAGT | Vada BAC sequence |
|  |  |  | *R:* CTCCCCAAGGCCAAGATAAGA |  |
| FQ2D6F2 | 65 |  | *F:* CCCGCCGGAATAGCAGAATCAGG | Vada BAC sequence |
|  |  |  | *R:* GCATCCGGCCACGTCCAGTCAG |  |
| FQ2D6F3 | 65 |  | *F:* CATCCGCGCAGCCACACCTTTCATA | Vada BAC sequence |
|  |  |  | *R:* ACATTTCCCGCCATTTCCGACAACT |  |
| FQ2D6F4 | 65 |  | *F:* TCTTGCATCTGGCGGAGGAACTG | Vada BAC sequence |
|  |  |  | *R:* TTTGGCACCGTATACCGAGGCTGAG |  |
| FQ2D7F2 | 65 |  | *F:* AGAAACTCCAACTCCTCGGCTCCAT | Vada BAC sequence |
|  |  |  | *R:* TGTCGACGCAATCTTAACCTTCTGA |  |
| FQ2D7F6 | 65 |  | *F:* GGATGCCATATTTCACGTAGACAGG | Vada BAC sequence |
|  |  |  | *R:* TCGTGGGAGGCATTGAGATTTGAGG |  |
| FQ2D8F6 | 65 |  | *F:* GTGACAACCGACCAACGAC | Vada BAC sequence |
|  |  |  | *R:* GCGAGCGCCTTATCCATTAG |  |
| FQ2D9F9 | 65 |  | *F:* GCGGGTAGGCCTTGGTCTGTTC | Vada BAC sequence |
|  |  |  | *R:* GGGAGGTGCATGCCAAAAAGTCAAT |  |
| FQ2D10F2 | 60 |  | *F:* CATGGCGGATTATTGGTGTTAGTAG | Vada BAC sequence |
|  |  |  | *R:* CAGTGCGGTGGGGTGCTC |  |
| P15M51-204 | 56 |  | *F:* CGGAGGAAACATGGACAACGAA | Marcel et al. (2007a) |
|  |  |  | *R:* AGCGAGCTCACTGCCAATCTACC |  |

| **Supplemental Table 8** Cont… | | | | |
| --- | --- | --- | --- | --- |
| Name | Tm  (°C) | Restriction  Enzyme | Primers sequences  (5’- 3’) | Source |
| Dominant markers amplifying Vada | | | | |
| *Rphq2.*V14 | 58 |  | *F:* CGCCGCCAACTGCAGCAAGAATCC | Vada annotated gene |
|  |  |  | *R:* CAACGTCGACGGCAGTCCCGATG |  |
| *Rphq2.*V16 | 58 |  | *F:* TTGCGGTGGAGTTCGACATCTTCA | Vada annotated gene |
|  |  |  | *R:* GTCATCGGGTCCACTTTGCCTTCC |  |
| *Rphq2.*V19 | 65 |  | *F:* CCCCGCGGTCTCATTCCTT | Vada annotated gene |
|  |  |  | *R:* TCTTTTTATCTTGGGCAACCGTGTA |  |
| *Rphq2.*V25 | 65 |  | *F:* TGTCTTCCTTCGGTTCCTTCC | Vada annotated gene |
|  |  |  | *R:* TCCGCCATGGCCACGATACG |  |
| Dominant markers amplifying SusPtrit | | | | |
| besS35P2K14EF | 58 |  | *F:* TTGAAACAGCTGGGGTCTT | BAC end |
|  |  |  | *R:* TGGTACACAAATATTCGTCTGC |  |
| FsQ2N2F3 | 56 |  | *F:* GCACGGGCGGCCACAGAGGAG | SusPtrit BAC sequence |
|  |  |  | *R:* TGTCGCCCAGCAGCTACGGAACC |  |
| FsQ2N2F8 | 62 |  | *F:* TGGCGGAGTCAAAATCAAGAGTT | SusPtrit BAC sequence |
|  |  |  | *R:* TCGTGGATATAGCGGCAGAGGTC |  |
| FsQ2N4F3 | 58 |  | *F:* GCTGATCCCACCCGCCATTC | SusPtrit BAC sequence |
|  |  |  | *R:* CATTCCTACCGCCCGCTTTCTTACG |  |
| FsQ2N5F5 | 63 |  | *F:* CCGCCGAGGACTGATACTT | SusPtrit BAC sequence |
|  |  |  | *R:* GCAACCAAACGCACCCTTAGA |  |
| FsQ2N11F6 | 62 |  | *F:* CACTTCTCCAATGACTGCCCTTATG | SusPtrit BAC sequence |
|  |  |  | *R:* ATCGCCTTTACGTGAACTATCCAG |  |
| FsQ2N11F8 | 58 |  | *F:* GAAATAATCAACTTGTGGCATAC | SusPtrit BAC sequence |
|  |  |  | *R:* CTTAGGGCAGCGAGGTTAG |  |
| FsQ2N11F9 | 62 |  | *F:* CATCATATTGGCAGCAGTGG | SusPtrit BAC sequence |
|  |  |  | *R:* AATCCCGAGCCTTCTTGACATA |  |
| FsQ2N12F3 | 63 |  | *F:* ACTGGTGGGTCCCCTTCTGGTA | SusPtrit BAC sequence |
|  |  |  | *R:* GCTTTGCCGGTCTTGTTCGTATT |  |
| FsQ2N13F2 | 63 |  | *F:* AGCCCCTCGACAGTTCCAGCATAGA | SusPtrit BAC sequence |
|  |  |  | *R:* CAGCCCGACCACATACCTCCACAGT |  |
| FsQ2N13F3 | 63 |  | *F:* AAAGAGGAGGGTGGCGGTGGTAGGA | SusPtrit BAC sequence |
|  |  |  | *R:* GGGGTGCTCGCGTCTGAACTCTGAA |  |
| FsQ2N13F9 | 60 |  | *F:* AGCGGTCTTAGTCTGGTCGTTGTA | SusPtrit BAC sequence |
|  |  |  | *R:* TCTTCAGGGCCATTTTCTATTTATC |  |

| **Supplemental Table 8** Cont… | | | | |
| --- | --- | --- | --- | --- |
| Name | Tm  (°C) | Restriction  Enzyme | Primers sequences  (5’- 3’) | Source |
| Dominant markers amplifying SusPtrit | | | | |
| FsQ2N13F10 | 62 |  | *F:* GGCCTCACTAACCAAAACGCAGAC | SusPtrit BAC sequence |
|  |  |  | *R:* ATGATTTTCCGACCACGACAACGAT |  |
| FsQ2N16F3 | 63 |  | *F:* GGGTGCTTGTGCCATGGGAGTAGG | SusPtrit BAC sequence |
|  |  |  | *R:* GGGGGTGGAGTGCGGAGGAAGAC |  |
| P14M54-252 | 56 |  | *F:* AGACCAGCATTACCTAAGCAGAGA | Marcel et al. (2007a) |
|  |  |  | *R:* AGAGGAGAGTGAGTGTAGGTGTCG |  |
| *Rphq2*.S01 | 58 |  | *F:* TGAAGGCGGGTTTGGTGTGGTGTA | SusPtrit annotated gene |
|  |  |  | *R:* CCCGCGTATGATTCTCTGCCTCTT |  |
| Co-dominant markers | | | | |
| *Rphq2*.V09 | 60 | *Nla*IV | *F:* GCCTCTACTTCCACGACTGC | Vada annotated gene |
|  |  |  | *R:* CCGGAGATGACGATGATGT |  |
| *Rphq2*.V30 | 65 | *Mbo*II | *F:* CGGCGGTGCGATCATAGAAT | Vada annotated gene |
|  |  |  | *R:* TCCCCGGCCGTAGAGTCC |  |
| *Rphq2.*V34 | 58 | *Taq*I | *F:* ACCCCGGCTCCCTCGTCCTC | Vada annotated gene |
|  |  |  | *R:* CTTTTGCCGCAGCGCCTTCATCT |  |
| S7300002F | 65 | *Sdu*I | *F:* GACGTTGAGGAGAGCAAAGG | SusPtrit BAC sequence |
|  |  |  | *R:* GCCGTTTATCACGAGGTTGT |  |

**Supplemental Table 8 Reference**

Marcel, T. C., R. Aghnoum, J. Durand, R. K. Varshney and R. E. Niks, 2007a Dissection of the barley 2L1. 0 region carrying the '*Laevigatum*' quantitative resistance gene to leaf rust using near-isogenic lines (NIL) and subNIL. Mol. Plant-Microbe Interact. **20:** 1604-1615.

| **Supplemental Table 9** Haplotypes of 194 barley accessions based on the presence (P) and absence (A) of V.Perox-3, S.UF and S.Kin-1. | | | | | |
| --- | --- | --- | --- | --- | --- |
| **Cultivars without V.Perox-3, and with S.UF and S.Kin-1 (= APP)** | |  | **Cultivars with V.Perox-3, and without S.UF and S.Kin-1 (= PAA)** | | |
| 116-5 | Impala |  | 17-5-16 | Extract | Potter |
| Abelone | Julia |  | Abed4611 | Ferment | Prestige |
| Akka | Kobinkatagi |  | Abed50015 | Fusion | Princesse |
| Allegro | L94 |  | Abed5193 | Gant | Prisma |
| Annasofie | Lamba |  | Adele | Gei | Prolog |
| Apex | Libelle |  | Alanis | Golden Promise | Punto |
| Aramir | Limbo |  | Albright | Goldie | Ricarda |
| Ardila | Lisbet |  | Alliot | H. laevigatum | Riga |
| Ark Royal | Lysiba |  | Annabell | Henni | Roxana |
| Belfor | Menuet |  | Aravis | Jacinta | Scarlett |
| Bella | Morex |  | Ariel | Jill | Senor |
| Berac | Mosane |  | Aspen | Korinna | SJ5085 |
| C92 | Nadrine |  | Astoria | Krona | SJ5095 |
| Calypso | NFC497-33 |  | Atem | Lenka | Steptoe |
| Caminant | Nudinka |  | Barke | Linus | Sultane |
| Caruso | Pirouette |  | Bartok | Lofa Abed | Texane |
| Cebada Capa | Polygena |  | Bereta | Loma | Thuringia |
| Chalice | Porthos |  | Bond | Lux | Tirup |
| Charon | Printa |  | Brenda | Lysimax | Tofta |
| Clermont | Proctor |  | Brewster | Mandolin | Trebon |
| CSBA 4374-11 | Prominant |  | Britta | Maud | Trianon |
| Delisa | Ragtime |  | C1 | Mazurka | UN AE 3.1 |
| Derkado | Ramona |  | C118 | Meltan | Vada |
| Diva | Rec |  | C123 | Mentor | Varunda |
| Dom | Reggae |  | Cadeau | Merete | Verona |
| Drossel | Rika |  | Canut | Midas | Viskosa |
| Effendi | Ruby |  | Caskant | Mie | Wren |
| Enigma | Shamu |  | Cathrine | Miralix |  |
| Franka | Steffi |  | Cecilia | Miranda |  |
| Freegold | Sultan |  | Century | Nevada |  |
| Georgie | SusPmur |  | Chamant | Nigrinudum |  |
| Gerkra | SusPtrit |  | Chariot | NSL 94-4109 |  |
| Gesine | Tamara |  | Christian | Optic |  |
| Give | Teal |  | Cicero | Optima |  |
| Gull | Tremois |  | Cooper | Otira |  |
| Gunhild | Trigo Biasa |  | Cork | P1391136 |  |
| Hanka | Tyra |  | Decanter | Paloma |  |
| Hassan | Valeta |  | Delibes | Pauline |  |
| Hellas | Vintage |  | Delita | Peel |  |
| Herta | Volla |  | Escort | PF11011-52 |  |
| Hydrogen | Zephyr |  | Etna | PF11202-53 |  |
| Igri |  |  | Evelyn | Pongo |  |
